# Vanadium Complexes with Thioanilide Derivatives of Amino Acids: Inhibition of Human Phosphatases and Specificity in Various Cell Models of Metabolic Disturbances

**DOI:** 10.3390/ph17020229

**Published:** 2024-02-09

**Authors:** Grzegorz Kazek, Monika Głuch-Lutwin, Barbara Mordyl, Elżbieta Menaszek, Monika Kubacka, Anna Jurowska, Dariusz Cież, Bartosz Trzewik, Janusz Szklarzewicz, Monika A. Papież

**Affiliations:** 1 Department of Pharmacological Screening, Faculty of Pharmacy, Jagiellonian University Medical College, Medyczna 9, 30-688 Krakow, Poland; 2 Department of Radioligands, Faculty of Pharmacy, Jagiellonian University Medical College, Medyczna 9, 30-688 Krakow, Poland; monika.gluch-lutwin@uj.edu.pl (M.G.-L.); barbara.mordyl@uj.edu.pl (B.M.); 3 Department of Cytobiology, Faculty of Pharmacy, Jagiellonian University Medical College, Medyczna 9, 30-688 Krakow, Poland; elzbieta.menaszek@uj.edu.pl; 4 Faculty of Pharmacy, Jagiellonian University Medical College, Medyczna 9, 30-688 Krakow, Poland; monika.kubacka@uj.edu.pl; 5 Coordination Chemistry Group, Faculty of Chemistry, Jagiellonian University, Gronostajowa 2, 30-387 Krakow, Poland; jurowska@chemia.uj.edu.pl (A.J.); szklarze@chemia.uj.edu.pl (J.S.); 6 Department of Organic Chemistry, Faculty of Chemistry, Jagiellonian University, Gronostajowa 2, 30-387 Krakow, Poland; ciez@chemia.uj.edu.pl (D.C.); trzewik@chemia.uj.edu.pl (B.T.)

**Keywords:** vanadium complexes, phosphatases, in vitro, cell models, metabolic disorders, diabetes, insulin resistance, gluconeogenesis, NAFLD

## Abstract

In the text, the synthesis and characteristics of the novel ONS-type vanadium (V) complexes with thioanilide derivatives of amino acids are described. They showed the inhibition of human protein tyrosine phosphatases (PTP1B, LAR, SHP1, and SHP2) in the submicromolar range, as well as the inhibition of non-tyrosine phosphatases (CDC25A and PPA2) similar to bis(maltolato)oxidovanadium(IV) (BMOV). The ONS complexes increased [14C]-deoxy-D-glucose transport into C2C12 myocytes, and one of them, VC070, also enhanced this transport in 3T3-L1 adipocytes. These complexes inhibited gluconeogenesis in hepatocytes HepG2, but none of them decreased lipid accumulation in the non-alcoholic fatty liver disease model using the same cells. Compared to the tested ONO-type vanadium complexes with 5-bromosalicylaldehyde and substituted benzhydrazides as Schiff base ligand components, the ONS complexes revealed stronger inhibition of protein tyrosine phosphatases, but the ONO complexes showed greater activity in the cell models in general. Moreover, the majority of the active complexes from both groups showed better effects than VOSO_4_ and BMOV. Complexes from both groups activated AKT and ERK signaling pathways in hepatocytes to a comparable extent. One of the ONO complexes, VC068, showed activity in all of the above models, including also glucose utilizatiand ONO Complexes are Inhibitors ofon in the myocytes and glucose transport in insulin-resistant hepatocytes. The discussion section explicates the results within the wider scope of the knowledge about vanadium complexes.

## 1. Introduction

Invariably, for years, type 2 diabetes (T2D) has been described as a global, social health problem. About 10% of the population suffer from diabetes, and the trend is still increasing. It is predicted that by 2035, there will be approximately 600 million diabetics worldwide [1]. A much broader and still growing health problem on a global scale consists of disorders of glucose and lipid metabolism with accompanying insulin resistance, which may predispose patients to overweight, obesity, dyslipidemia, and glucose metabolism disturbances [2,3,4]. 

Altered glucose and lipid metabolism are also key factors associated with the development of nonalcoholic fatty liver disease (NAFLD), i.e., following the recently proposed nomenclature, metabolic-dysfunction-associated fatty liver disease. NAFLD is the most common liver disorder, present in approximately 25% of the population, and it is estimated that NAFLD will be the most common indication for liver transplantation by 2030 [5]. Currently no therapies are registered for the treatment of NAFLD [6]. 

In T2D pharmacotherapy, 56 antihyperglycemic drugs have been approved as monotherapies and combination therapies by the Food and Drug Administration. Despite the wide spectrum of pharmacotherapy options, a large proportion of patients have difficulties in achieving sufficient clinical improvement and reducing the risk of T2D complications. This explains the presence of almost 100 additional antihyperglycemic drugs currently being evaluated in clinical trials [7,8]. And although vanadium compounds are not included among the main pharmacotherapeutic groups used to treat metabolic disorders, their more than 100 year history of clinical trials and a wide range of studies indicate their potential for anti-diabetic activity [9,10,11,12].

Many of vanadium compounds’ mechanisms of action are similar to the mechanisms of action of registered antidiabetic drugs, as well as potential pharmacotherapeutic targets in glucose and lipid metabolism disorders [13,14,15]. These common pharmacological mechanisms of vanadium’s effects include increasing glucose uptake into cells, improving insulin sensitivity, and enhancing insulin action, involving target tissues and organs: adipose tissue, muscle, and the liver [16]. Skeletal muscles are the main organ responsible for glucose homeostasis in the body [17,18]. Like skeletal muscle, adipose tissue and the liver play key roles in lipid and glucose metabolism, and metabolic impairment of adipocytes and hepatocytes is associated with metabolic disorders [19]. Currently available pharmacological interventions in metabolic diseases target these three tissues [19,20]. 

The main and best-studied intracellular target for vanadium compounds is the protein tyrosine phosphatase 1B (PTP1B), and their inhibition allows the insulin receptor to remain activated, thereby potentiating the effect of endogenous insulin [21]. This leads to the activation of the intracellular signal pathway, the AKT and PI3K kinases (phosphatidylinositol 3-kinase), which are considered potential therapeutic targets for diabetes and other conditions associated with insulin resistance [13,22,23]. The molecular mechanism responsible for the insulin-like effects of vanadium compounds has been shown to involve the activation of several key components’ intracellular signaling pathways, including, among other things, the mitogen-activated protein kinases (MAPKs) and extracellular-signal-regulated kinase 1/2 (ERK1/2) [22,24,25]. These pathways are essential for controlling energy homeostasis and are related to insulin sensitivity, glucose tolerance, hepatic steatosis, obesity, and diabetes. 

Vanadium compounds are also inhibitors of other phosphatases besides PTP1B that are fundamental for the function of many metabolic pathways and transcription factors with wide implications for many metabolic disorders [16,21,26,27,28]. One example consists of the Src homology-2-domain-containing phosphatases type 1 and 2 (SHP1 and SHP2) and the leukocyte-common-antigen-related (LAR) [29]. The receptor protein tyrosine phosphatase alpha (RPTPA) also has described implications in insulin action [30]. Vanadium compounds have been shown to inhibit not only protein tyrosine phosphatases but also serine/threonine phosphatase activity, e.g., protein phosphatase 2A (PP2A) [31,32]. These phosphatases are also promising targets for drug development in diabetes treatment [33,34]. In addition, vanadium compounds are inhibitors of dual-specific phosphatases including cell division cycle 25 A phosphatase (CDC25A), which is one of the most crucial cell cycle regulators [35,36].

The above-discussed targets and molecular mechanisms of action of vanadium may result in non-specific and multidirectional biological and pharmacological effects. Other difficulties in the development of pharmaceuticals based on vanadium also result from its high chemical reactivity in bodily fluids as well as speciation and the lack of stable metabolites [15]. The chemical affinity of vanadium for oxygen, nitrogen, or sulfur atoms, as well as its flexibility in coordination geometry, allows it to form stable complexes or transition state complexes with many biological molecules and affects biological activity [37,38]. It is because of these chemical properties that the search for pharmacologically active, new vanadium compounds in the form of its complexes is such a promising, main direction of research for the use of vanadium in diabetes and other diseases. Of particular importance here is the advantage of complexes over organic compounds resulting from the variability of coordination numbers, geometry, redox states, ligand substitutions, and structural diversity [9,39]. The aim of modern research in the development of pharmaceuticals based on vanadium compounds is the design, synthesis, and biological testing of organic ligands or chelators with improved properties [40,41]. A difficult task that may yield great benefit is the development of vanadium complexes with greater specificity for biological and therapeutic targets.

Previous research by our team on vanadium complexes with potential application in metabolic disorders involved the synthesis of over a hundred new vanadium complexes from different structural groups, which have been described in publications (e.g., [42,43,44,45,46,47,48,49,50,51,52]) and patented in part [52,53]. For the above-mentioned 110 vanadium complexes, screening tests for antidiabetic activity in vitro and in three cell-based models were conducted, making our research unique and significant [54]. This allowed for the selection of the most active new vanadium complexes as potential compounds for metabolic disorders. Among these complexes were the five vanadium complexes with thioanilide amino acid derivatives (ONS complexes), synthesized and described for the first time, which are the subject of this publication.

The aim of this study was to evaluate the pharmacological activity of the newly synthesized ONS complexes and their effects on the metabolic processes associated with various disturbances of glucose and lipid metabolism. 

**Scheme 1:**
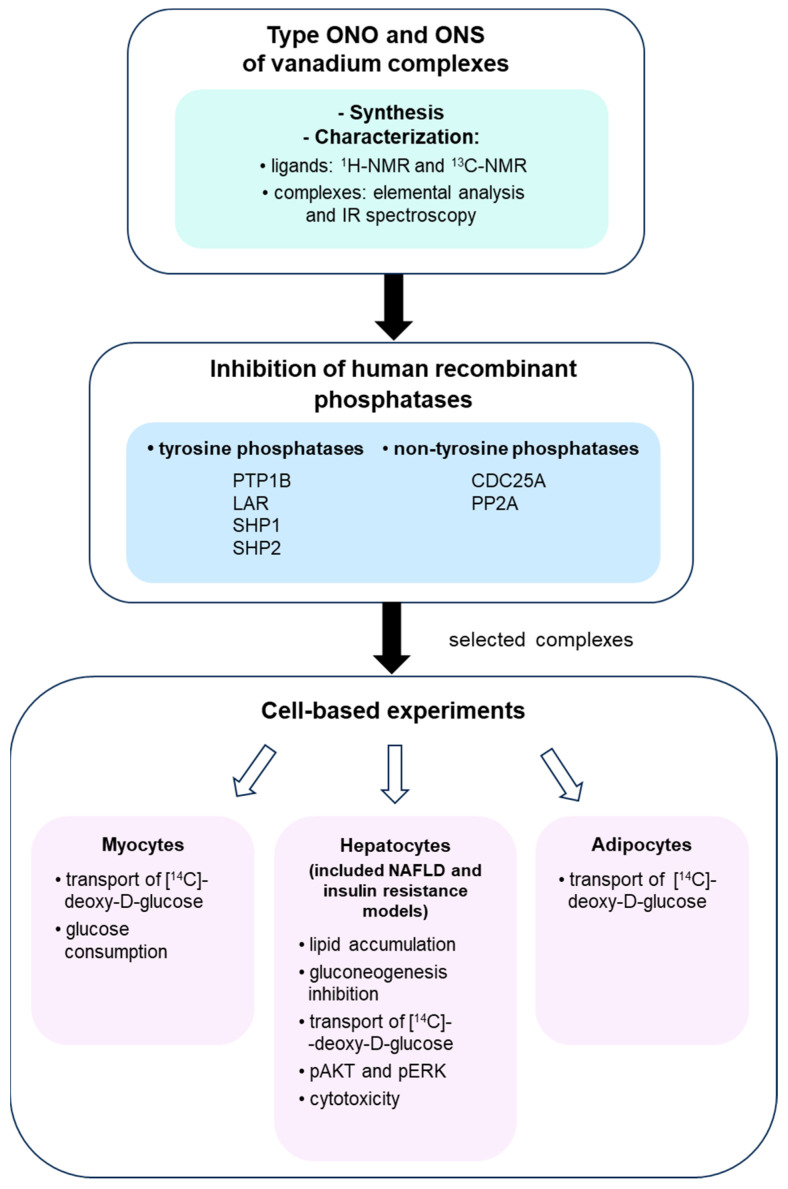
The workflow of the study.

In this paper, the synthesis and the physical and chemical characteristics of the novel vanadium complexes with ONS ligands are described (the workflow of the study is presented on the Scheme 1). For these complexes, the inhibition of human PTPs and non-tyrosine phosphatases was studied. In myocytes and adipocytes, the uptake of radiolabeled glucose analog was evaluated. Additionally, the effect of vanadium complexes on glucose metabolism was also investigated by assessing glucose consumption in the skeletal muscle cell model. Moreover, the accumulation of lipids in the cell model of non-alcoholic fatty liver disease (NAFLD) and the inhibition of gluconeogenesis in hepatocytes were studied. Experiments were conducted to assess the effect of vanadium complexes on radiolabeled glucose uptake to hepatocytes, in which insulin resistance was induced by the pro-inflammatory factor TNF and hyperlipidemia. Finally, the effect on radiolabeled glucose transport under hyperinsulinemia conditions was also tested, and the influence of vanadium complexes on the activity of the cellular kinases, AKT and ERK, in hepatocytes were carried out. 

To compare the biological activity of the ONS complexes, we also conducted the tests mentioned above for the vanadium ONO complexes described previously [45,46,47,55]. Compared to previously published results for these ONO complexes, the scope of research has been extended. Such an approach to the subject allowed for broader conclusions regarding the influence of the structure of vanadium complexes on biological activity.

Therefore, this study represents a continuation of our studies that used a unique screening approach to test the activity of vanadium complexes. The broad range of models and research methods used in comparison to our previous studies and those of other authors constitutes the novelty of this research.

## 2. Results

### 2.1. Synthesis and Characteristics of the ONS and ONO Complexes

**Scheme 2:**
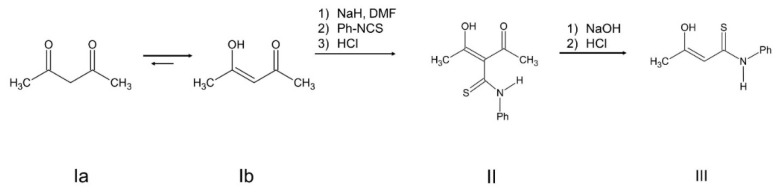
Two-step synthesis of 3-hydroxytiocrotonic acid anilide (III) as the starting compound for the preparation of ONS ligands.

**Scheme 3:**
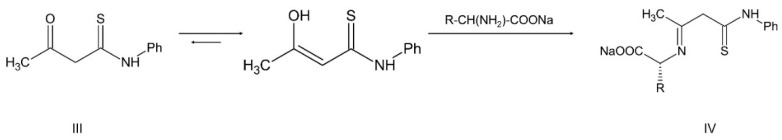
Condensation reaction of a thioanilide with the sodium salt of an amino acid, leading to obtaining a ligand IV with an imine structure (possible imine-enamine tautomerism). R = L-tryptophan, L-phenylalanine, L-leucine, L-methionine, or D/L-isoleucine.

**Scheme 4:**
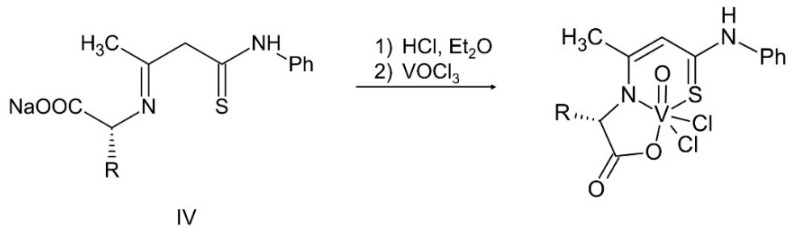
Synthesis of the complexes with the ONS ligands. R = L-tryptophan, L-phenylalanine, L-leucine, L-methionine, or D/L-isoleucine.

The synthesis and characterization of the ONS complexes is presented for the first time (Scheme 2, Scheme 3 and Scheme 4, Table 1). The 3-hydroxytiocrotonic acid anilide (III), as the starting compound for the preparation of ONS ligands, was obtained in a two-step synthesis according to Scheme 2. In the first stage, the acetylacetone in the reaction with phenyl isothiocyanate gave intermediate 2-acetyl-3-hydroxythiocrotonic acid anilide (II), which was then deacetylated to the desired 3-hydroxytiocrotonic acid anilide (III). In the next stage, the condensation reaction of (III) with appropriate L-amino acids sodium salts took place, yielding the ONS Schiff base ligands (IV) (Scheme 3). Finally, the vanadium complexes with the ONS ligands (IV) were synthetized in the reaction with vanadium (V) oxychloride (VOCl_3_) (Scheme 4).

For the obtained ONS vanadium complexes (VC054, VC059, VC070, VC073, and VC109), the formulas are presented in Table 1. These formulas are based on the results of the elemental analysis and infrared spectra (see also the Supplementary Materials). 

Syntheses of these complexes were repeated many times; their analyzes and IR spectra were reproducible within error limits, which proves the purity of the obtained com-pounds and the repeatability of the syntheses.

Moreover, the NMR spectra (^1^H-NMR as well as ^13^C-NMR with signal assignment) of L_1_–L_5_ confirmed the formation of ONS-type ligands (see the Supplementary Materials). The NMR spectra of ligands showed that most of the ligands, except L_1_ and L_4_, were in the imine form. In the case of L_1_ and L_4_, however, the imine-enamine tautomerism was quite evident in the ^1^H-NMR spectra, e.g., for L_4_, there was a small signal at 5.36 ppm from the =CH- proton, which was present in place of the CH_2_ protons. In addition, some other signals were doubled, which was also indicated in the description of the signal assignment. This was particularly evident in the CH_3_ group signal, which was doubled at 2.107 and 2.098 ppm and in the SCH_2_ group at 2.71 and 2.60 ppm (where there were two triplets with a mutual ratio of 4:1). This interpretation was presented under the spectra. In the ^13^C-NMR spectra of the L_4_ ligand, there were also some additional signals from the enamine form, but they were not as clear as in the proton spectrum.

We could not obtain a crystal suitable for single crystal X-ray measurement as the crystals formed were too small. All complexes were diamagnetic, which confirmed the presence of vanadium (V). To the metal center, two chlorido ligands, one oxido, and one ONS organic ligand were coordinated. The coordination sphere of vanadium in ONS-type complexes may not be crucial for biological studies, as in the body’s environment, the chlorido ligands are labile.

The ONS complexes are soluble in water and organic solvents. This may be related to the presence of chloro ligands that, when detached, can lead to the formation of an ionic complex that will be more soluble. Moreover, an increase in the ionic radius of the sulfur atom, in comparison to that of the oxygen atom, and the presence of two chloro ligands can cause stress in the coordination sphere of vanadium, leading to deformation of the structure and easier detachment of ligands, which leads to the formation of cationic complexes. The stability of the ONS complexes was tested in DMSO/H_2_O (20 μL+3 mL), and DMSO was the solvent for preparing the stock solution in pharmacological tests. Besides pH = 7, the stability was tested at the pH = 2 condition. These complexes showed stability in this condition and at the time of incubation. Their UV–VIS spectra in solution are attached in the Supplementary Materials. The positions of the bands were very similar to the spectra in the DMSO itself.

**Figure 1:**
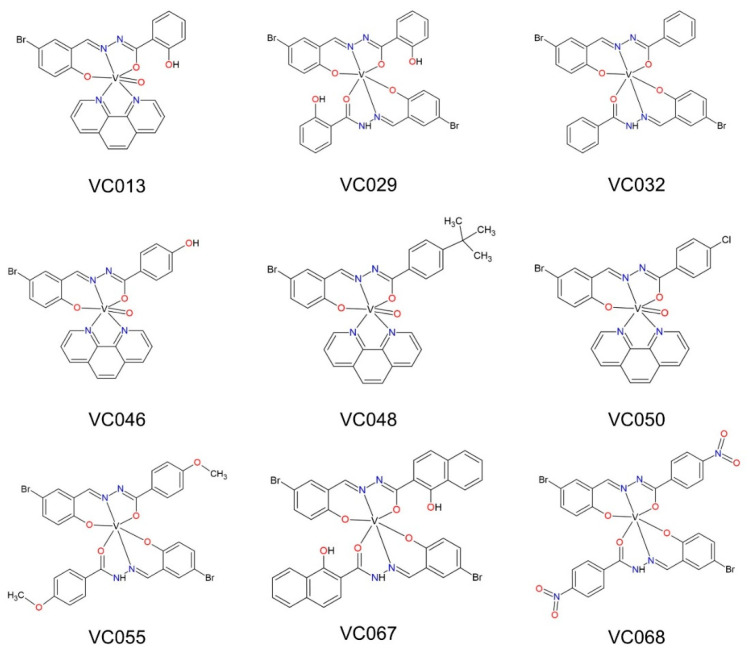
The structural formulas of the ONO complexes.

In our previous studies, we described the ONO vanadium complexes. The series of the ONO vanadium complexes have been characterized in references [45,46,47]. Only the complex denoted as VC055 was not described; therefore, we present the synthesis and physicochemical characterization in Section 4. The formulas of the vanadium complexes, the components of Schiff base ligands (used in syntheses), and the literature references are given in Figure 1 and Table 2. We selected complexes with 5-bromosalicylaldehyde and substituted benzhydrazides as components of the Schiff base ligand. For VC013, VC046, VC048, and VC050 complexes, 1,10-phenantroline (phen), as a co-ligand, stabilized V(IV). For V(III) complexes (VC029, VC032, VC055, VC067, and VC068), two Schiff base ligands compensated the charge of the metal center. These complexes were stored under argon, but after a longer time, they were oxidized to V(IV) without changing the composition of the compound, as described in our previous publications [46].

### 2.2. The ONS Complexes Inhibited the Activity of Human Tyrosine Phosphatases Stronger Than the ONO Complexes

At first, in our screening tests, we showed that ONS complexes, as well as ONO ones, at a concentration of 1 µM, inhibited human tyrosine phosphatases PTP1B, LAR, SHP1, and SHP2; however, ONS complexes exhibited higher activity than ONO ones (Table 3). ONS complexes inhibited LAR phosphatase activity in the range of 56 to 69% relative to controls (100% activity), and the percentage inhibition of the other phosphatases was similar, ranging from 70 to 88%. Most of the ONO complexes showed weaker inhibitory potency than the ONS complexes and comparators. 

The ONO complexes exhibited significantly greater diversity in the strength of inhibition of protein tyrosine phosphatases than the ONS complexes. For instance, the highest values of PTP1B inhibition were shown by the VC013 complex, which inhibited the activity of this phosphatase by 60%, and the VC032 by 9% was the weakest complex in the ONO group (Table 3). Aside from VC013, the other ONO complexes demonstrated higher inhibitory potency than VOSO_4_ and BMOV. The last two were used as comparator vanadium compounds to evaluate the relative activity of the tested complexes. Suramin and ammonium heptamolybdate ((NH_4_)_6_Mo_7_O_24_) were used as control compounds, organic and non-vanadium inorganic inhibitor of PTPs respectively.

In the next step, we confirmed the effects observed in the screening tests by determining the IC50 for selected complexes from both groups (Table 4). To determine the IC50, the ONO complexes that had shown significant biological activity in our previous studies were selected [46], despite not demonstrating the highest inhibition in screening assays. For all tested phosphatases, the IC50 values obtained for ONS complexes were similar to IC50 values for VOSO_4_ and BMOV. However, ONO complexes showed approximately 30–40 times weaker inhibition than ONS complexes and comparators (Table 4). Ammonium heptamolybdate ((NH_4_)_6_Mo_7_O_24_) was used as a non-vanadium, inorganic control compound.

### 2.3. The ONS and ONO Complexes Are Inhibitors of Non-Tyrosine Phosphatases

The ONS and ONO complexes also showed inhibition of non-tyrosine phosphatases: CDC25A dual specificity phosphatase and PPA2 serine-threonine phosphatase in a range similar to that of VOSO_4_ and BMOV (Table 5). The exception was VC050, which at the concentration of 1 µM showed weaker inhibition than the other complexes and comparators. VC050 also showed less activity on the inhibition of tyrosine phosphatases, as shown above.

### 2.4. The ONS Complexes Enhanced Glucose Transport into Myocytes and Adipocytes to a Lesser Extent Than ONO Complexes

**Figure 2:**
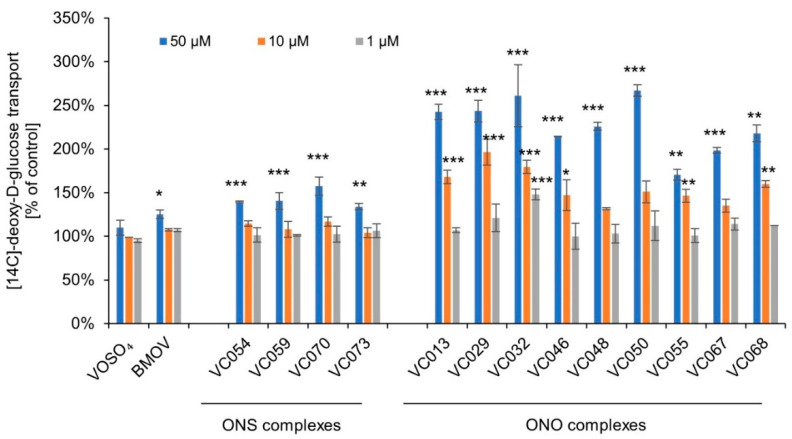
The effect of vanadium complexes on the transport of [14C]-deoxy-D-glucose into myocytes C2C12. The results are presented as a percentage of the control cells treated with the solvent only (mean ± SD). Statistical significance vs. control is denoted as * *p* ≤ 0.05, ** *p* ≤ 0.01, *** *p* ≤ 0.001. Results for VC029, VC046, VC050, VC067, and VC068 were previously published by us in [46].

At the concentration of 50 µM, ONS complexes caused an increase in the transport of [14C]-deoxy-D-glucose into C2C12 myocytes in the range of 119–158% of the control. VOSO_4_ and BMOV showed an increase in transport to 110% and 125% of the control, respectively, with statistical significance only for BMOV (Figure 2). 

On the other hand, the observed effect of ONO complexes on [14C]-deoxy-D-glucose transport was significantly greater than that of ONS complexes. ONO complexes significantly enhanced transport, from 199% to 267% of the control, with statistical significance between *p* ≤ 0.001 and *p* < 0.05. VC032, the most active complex at the concentration of 50 µM, also showed significant effects at concentrations of 10 and 1 µM, indicating a concentration-dependent response. 

**Figure 3:**
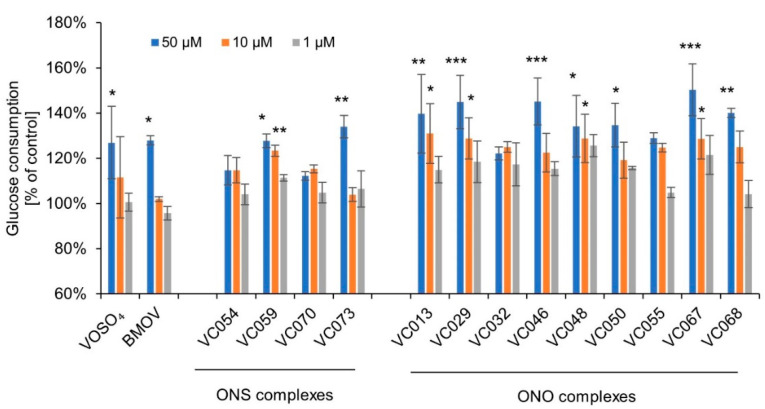
The effect of vanadium complexes on glucose consumption by myocytes C2C12. The results are presented as a percentage of the control cells treated with the solvent only (mean ± SD). Statistical significance vs. control is denoted as * *p* ≤ 0.05, ** *p* ≤ 0.01, *** *p* ≤ 0.001.

At the highest tested concentration, the ONS complexes increased glucose consumption by myocytes C2C12 in the range 104 to 134% of the control value (Figure 3). 

ONO complexes at the concentration of 50 µM increased glucose consumption within a range of 122 to 150% compared to the control (*p* ≤ 0.001 to 0.05). No statistically significant effect was observed only for VC032 and VC055.

Simultaneously with glucose consumption tests, the viability of myocytes C2C12 was examined due to their prolonged 24 h incubation with vanadium complexes. Cell viability was not lower than 80% of the control (solvent only) for all tested complexes, which is recognized as a lack of cytotoxic effect [56]. After incubation with the tested complexes at a concentration of 50 µM, the lowest viability was observed for VC054 (84 ± 8%) and VC059 (86 ± 6%), while it was 90 ± 4% for BMOV. For some complexes, as well as the rosiglitazone used as a reference compound, an increase in the signal was observed (e.g., VC055 127 ± 5% and rosiglitazone 120 ± 7%) [47].

**Figure 4:**
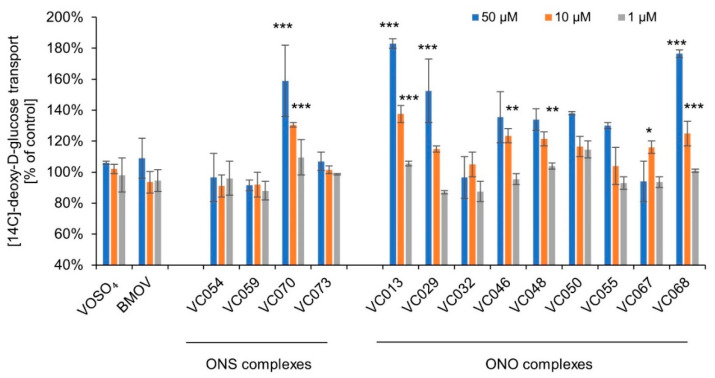
The effect of vanadium complexes on the transport of [14C]-deoxy-D-glucose into adipocytes 3T3-L1. The results are presented as a percentage of the control cells treated with the solvent only (mean ± SD). Statistical significance vs. control is denoted as * *p* ≤ 0.05, ** *p* ≤ 0.01, *** *p* ≤ 0.001. Results for VC029, VC046, VC050, VC067, and VC068 were previously published by us in [46].

Among the ONS complexes, only VC070 enhanced the transport of [14C]-deoxy-D-glucose into 3T3-L1 adipocytes (159% of control at 50 µM, *p* ≤ 0.001; Figure 4). This complex also exhibited the strongest effect on radiolabeled deoxy-D-glucose transport into C2C12 myocytes. The remaining ONS complexes did not significantly affect this transport (92–107% of control at 50 µM, *p* > 0.05). Similarly, the comparators BMOV and VOSO_4_ showed low activity in comparison to the control.

For the ONO complexes, two of them did not affect the transport of [14C]-deoxy-D-glucose into 3T3-L1 adipocytes: VC032 and VC067 (Figure 4). The rest of the complexes in this group intensified this transport in a range of 134 to 177% of the control, with statistically significant activity at a concentration of 50 µM observed for complexes VC013, VC029, and VC068 (*p* ≤ 0.001). 

It is noteworthy that both groups of complexes exhibited differential effects on the transport of [14C]-deoxy-D-glucose in the above cellular models (myocytes vs. adipocytes). Among ONS complexes, only VC070 showed activity in both models (myocytes C2C12 158 ± 10% and adipocytes 3T3-L1 159 ± 14% of the control). The rest of the complexes in this group did not demonstrate significant activity in adipocytes 3T3-L1 (92%–107% of control, *p* > 0.05). The ONO complexes VC032 and VC067 enhanced the transport of [14C]-deoxy-D-glucose only in myocytes C2C12, without exhibiting similar activity in adipocytes 3T3L-1. VC032 caused an increase in this transport into myocytes (266 ± 36% of control) and no effects in adipocytes (97 ± 14%; *t*-test, *p* = 0.04). For VC067, these values were 199 ± 3% and 94 ± 14% for myocytes and adipocytes, respectively (*t*-test, *p* = 0.0002). The differences in the activity of the other tested ONO complexes did not show statistically significant differences (*t*-test, *p* > 0.05).

### 2.5. The ONO Complexes but Not the ONS Complexes Reduced Hepatocyte Steatosis in the Cellular Model of Non-Alcoholic Fatty Liver Disease (NAFLD)

**Figure 5:**
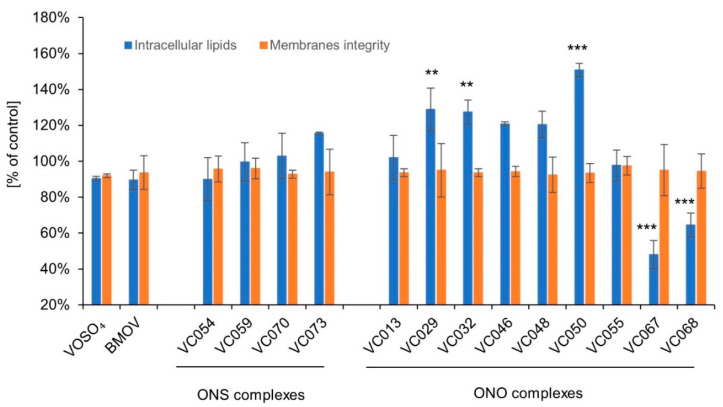
The effect of incubation of the vanadium complexes (10 µM) during induction steatosis on the accumulation of intracellular lipid and cytotoxicity in hepatocytes HepG2. The results are presented as a percentage of the control cells treated with the solvent only (mean ± SD). Statistical significance vs. control is denoted as, ** *p* ≤ 0.01, *** *p* ≤ 0.001.

In the cellular model of hepatic steatosis, the ONS complexes showed no effect on the amount of lipid accumulation after the induction of hepatocyte steatosis with oleic acid (90–116% of control, *p* > 0.05; Figure 5). Similarly, the comparators VOSO_4_ and BMOV did not have any significant effect in comparison to the control. 

Two ONO complexes showed very distinct effects. The accumulations of lipids in hepatocytes were 52% and 36% lower for VC067 and VC068, respectively, compared to the control (*p* ≤ 0.001) (Figure 5). The ONO complexes VC029, VC032, and V050 caused a large and statistically significant increase in lipid accumulation in hepatocytes (from 128% to 151% of the control, *p* ≤ 0.001 for VC050 and *p* ≤ 0.01 for the other two complexes). This may have been due to an undesirable effect on metabolic mechanisms or a hepatotoxic effect resulting in hepatocyte steatosis, without damage or reducing the number of hepatocytes [57].

We conducted cytotoxicity studies on the same hepatocytes used to measure lipid accumulation after incubation with the tested complexes. We also conducted simultaneous analysis of cell membrane integrity. Assays showed no significant differences from controls for any of the tested ONS and ONO complexes; therefore, the effect of reducing intracellular lipids should not be attributed to a reduction in cell number due to a cytotoxic effect. 

### 2.6. The ONS and the ONO Complexes Inhibited Gluconeogenesis in Hepatocytes

Continuing the research on the effects on the hepatic mechanisms of metabolic disorders, studies were conducted on the effect of selected ONS and ONO complexes on gluconeogenesis in HepG2. Among them were the ONO complexes VC067 and VC068, which showed inhibition of lipid accumulation in the same cells.

**Figure 6:**
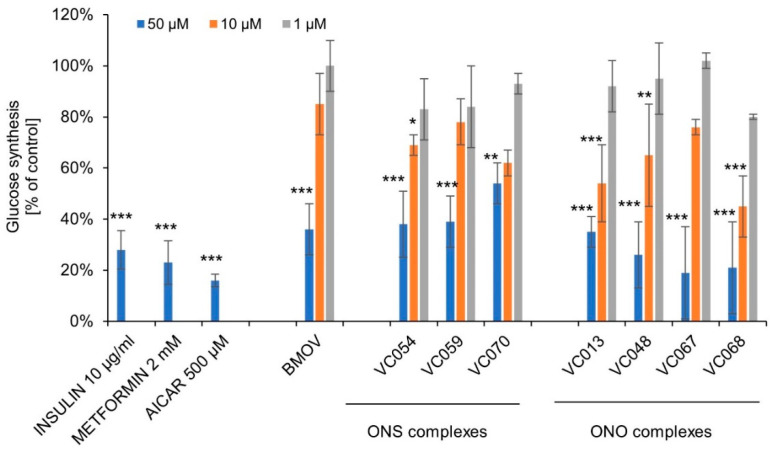
The effect of the vanadium complexes on gluconeogenesis in hepatocytes HepG2. The results are presented as a percentage of the control cells treated with the solvent only (mean ± SD). Statistical significance vs. control is denoted as * *p* ≤ 0.05, ** *p* ≤ 0.01, *** *p* ≤ 0.001.

All the tested complexes of both types and BMOV at the concentration of 50 µM showed potent and statistically significant inhibition of glucose synthesis in hepatocytes, ranging from 46 to 81% of the control (Figure 6). Known inhibitors of gluconeogenesis, metformin (a first-line treatment for type 2 diabetes), insulin, and AICAR (5-aminoimidazole-4-carboxamide riboside) as an AMPK activator inhibiting transcription of the gluconeogenesis genes, were used as the experimental control. All the control compounds showed inhibition of glucose synthesis by hepatocytes.

### 2.7. The ONO Complexes Reversed the Impairment of Glucose Transport to Hepatocytes under Conditions of Insulin Resistance and Hyperinsulinemia

**Figure 7:**
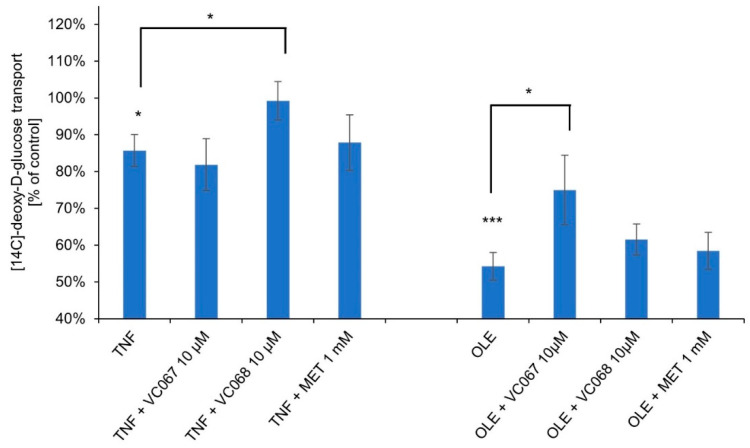
The effect of vanadium complexes on the transport of [14C]-deoxy-D-glucose into hepatocytes HepG2, in which insulin resistance was induced by preincubation with TNF and oleic acid (OLE). MET—metformin. The results are presented as a percentage of the control cells treated with the solvent only (mean ± SD). Statistical significance is denoted as * *p* ≤ 0.05, *** *p* ≤ 0.001. For TNF and OLE statistical significance vs. control cells.

Insulin resistance of HepG2 hepatocytes was induced in two ways: by preincubating them with the proinflammatory cytokine TNF and with oleic acid, which in both cases resulted in a decrease in [14C]-deoxy-D-glucose transport (Figure 7). Then, these hepatocytes were incubated with the ONO complexes VC067 and VC068 that most effectively among the tested complexes reduced lipid accumulation and inhibited gluconeogenesis in the same cells.

At the concentration of 10 µM, the VC067 complex reversed the decrease in [14C]-deoxy-D-glucose transport in hepatocytes in which insulin resistance was induced by oleic acid, and the VC068 complex reversed the decrease in transport induced by TNF (*p* ≤ 0.05). The observed effects were greater than those for 1 mM metformin.

**Figure 8:**
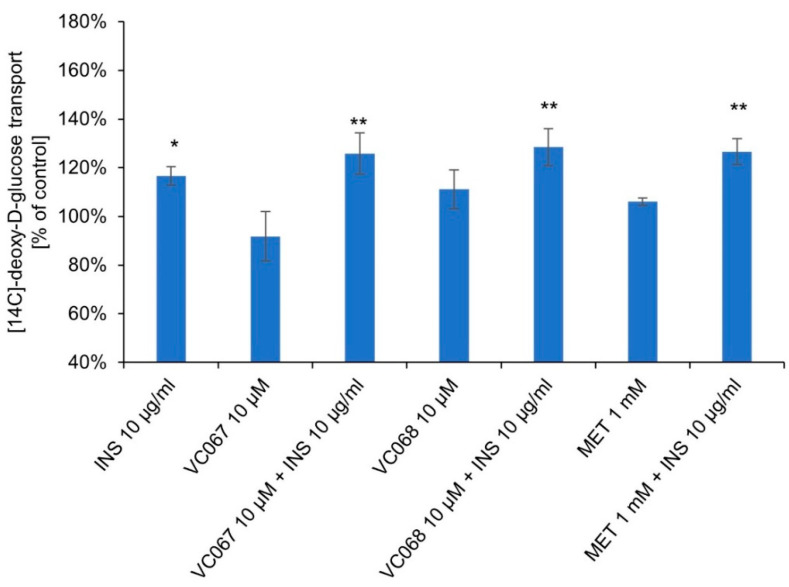
The effect of vanadium complexes on the transport of [14C]-deoxy-D-glucose into hepatocytes HepG2, in which insulin resistance was induced by preincubation with insulin (INS). CON—control, MET—metformin. The results are presented as a percentage of the control cells treated with the solvent only (mean ± SD). Statistical significance vs. control is denoted as * *p* ≤ 0.05, ** *p* ≤ 0.01. For insulin statistical significance vs. control insulin-resistant cells.

The complexes did not potentiate the transport of [14C]-deoxy-D-glucose to hyperinsulinemia-induced insulin-resistant hepatocytes at 10 µM, nor did metformin at 1 mM (Figure 8). Insulin resistance of hepatocytes was only reversed by very high concentrations of insulin (10 µg/mL). 

### 2.8. The ONS and ONO Complexes Activated ERK and AKT Signaling Pathways

**Figure 9:**
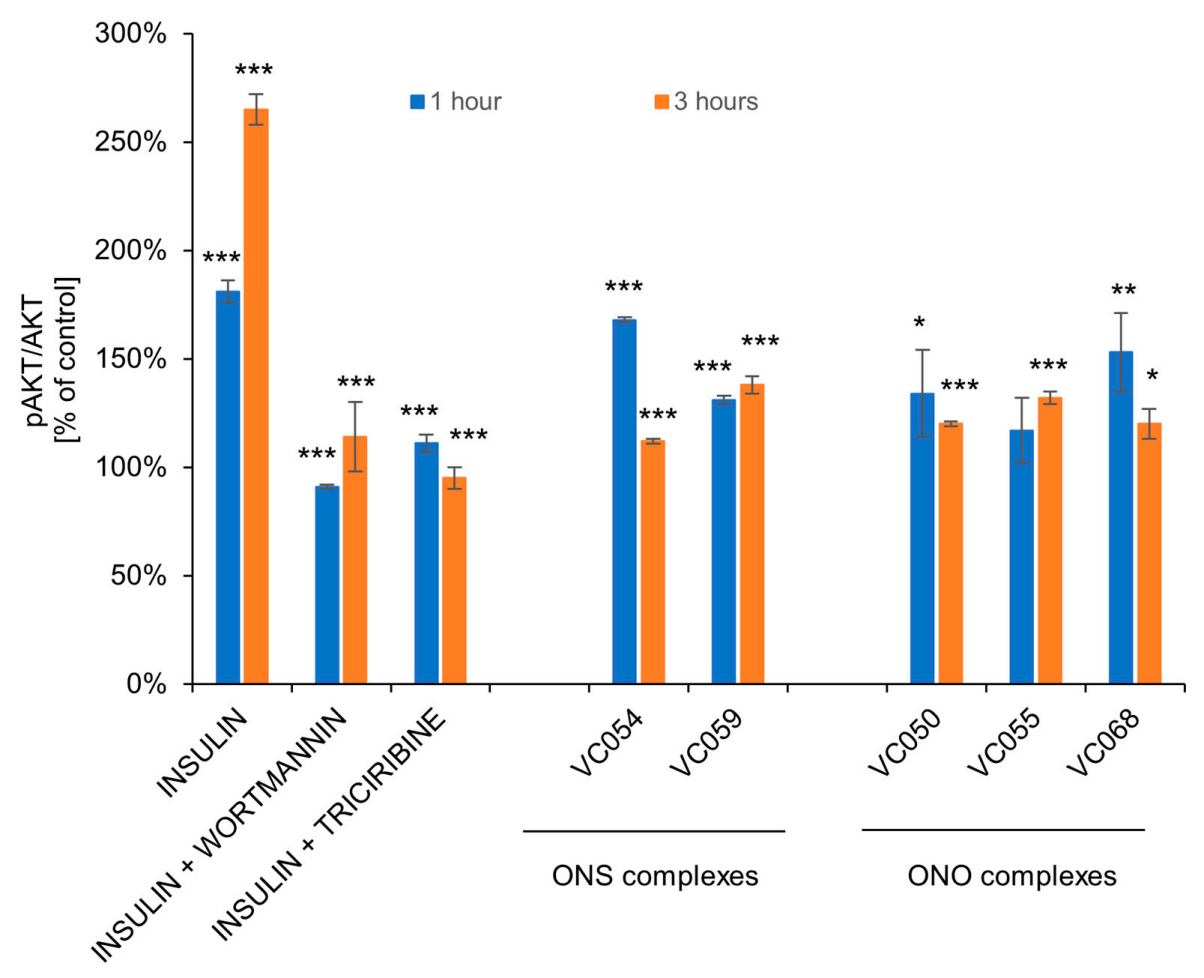
The effect of the vanadium complexes on AKT(Thr308) phosphorylation in HepG2 hepatocytes. The results are presented as a percentage of the pAKT/AKT ratio against control cells treated with the solvent only (mean ± SD). Human insulin at a concentration of 10 µg/mL, wortmannin at 1 µM, and triciribine at 10 µM were used as the controls of the experimental model. Statistical significance vs. control is denoted as * *p* ≤ 0.05, ** *p* ≤ 0.01, *** *p* ≤ 0.001. For insulin with AKT inhibitors, statistical significance vs. insulin alone.

For the selected ONS and ONO complexes, studies on the effect on the phosphorylation of AKT and ERK kinases as molecular targets of insulin and vanadium compounds were performed. Under the influence of the tested complexes at a concentration of 50 µM, an increase in the level of AKT phosphorylation in HepG2 hepatocytes was observed, in the range of 117–168% of the control after 1 hour and in the range of 112–138% of the control 3 h after the end of cell exposure (Figure 9). Human insulin was used as the experiment control for AKT activation. Wortmannin, a PI3K inhibitor, and triciribine, a highly selective AKT inhibitor, were used for inhibition of insulin effects on AKT.

**Figure 10:**
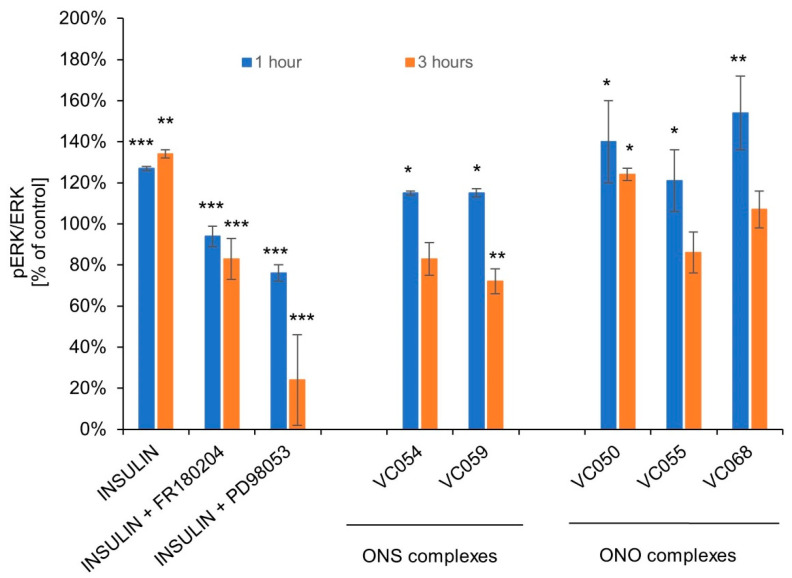
The effect of the vanadium complexes on ERK1/2 (Thr202/Tyr204) phosphorylation in HepG2 hepatocytes. The results are presented as a percentage of the pERK/ERK ratio against control cells treated with the solvent only (mean ± SD). Human insulin was used at a concentration of 10 µg/mL, FR180204 at 2 µM, and PD98053 at 50 µM. Statistical significance vs. control is denoted as * *p* ≤ 0.05, ** *p* ≤ 0.01, *** *p* ≤ 0.001. For insulin with ERK inhibitors, statistical significance vs. insulin alone.

All of the tested complexes also enhanced ERK phosphorylation. Human insulin was used as positive control for ERK phosphorylation. FR180204, a selective ERK inhibitor, and PD98053, an MEK inhibitor, were used as control compounds for the inhibition of the insulin effects (Figure 10).

## 3. Discussion

Most studies have analyzed the antidiabetic effects of vanadium complexes in a narrow and limited scope, despite the complex mechanisms underlying this activity. Only a wide range of studies can reveal the full potential of the therapeutic possibilities of vanadium complexes, and this is the approach we used in our work. We conducted in vitro studies of human phosphatase inhibition, and we used various cell models of organs and tissues connected with the pathogenesis of metabolic disorders: myocytes, adipocytes, and hepatocytes. In these models, we studied numerous processes related to the pharmacotherapy of metabolic disorders: glucose transport and its utilization, as well as lipid accumulation, gluconeogenesis, and insulin resistance in hepatocytes. Importantly, we studied the effects of vanadium targeting not only diabetes itself, but also other metabolic disorders associated with or leading to diabetes.

Searching for new vanadium complexes with better properties and biological effectiveness, we synthesized new complexes with thioanilide amino acid derivatives. We decided to use ONS ligands, comparing them with ONO ligands to check whether the change of one oxygen into one sulfur atom in the first coordination sphere influences biological activity. To have very similar complexes, five- and six-membered rings should be formed. Therefore, we used thioanilide as a six-membered component and an analogue of the salicylaldehyde used in ONO-type complexes. Amino acids were used due to the presence of the NH_2_ group that can form an imine bond with the carbonyl group of the thioanilide. Additionally, a COO^−^ group of amino acids has a donor oxygen atom in the appropriate position to form a five-membered ring with a central vanadium atom. Moreover, the presence of sodium salts formed of amino acids increased the solubility of the obtained vanadium complexes, which is important from the point of view of the compound’s bioavailability. When comparing the biological activity of complexes with ONO- and ONS-type ligands, the presence of a more polarizable sulfur atom, compared to the oxygen atom, leads to the stabilization of the fifth oxidation state of vanadium. The influence of the substituents in the ONS Schiff base ligand on biological activity is not as significant as in the case of the ONO-donor ligands. The results of pharmacological tests for the ONS complexes are more comparable within this group, in contrast to the ONO complexes. 

Despite orthovanadate (VO_4_^3−^) being present in aqueous media in the form of H_2_VO_4_^−^ and HVO_4_^2−^, uncomplexed vanadium (VO_4_^3−^) binds in the active site of enzymes and is considered as a biologically active part of vanadium complexes and other organovanadium compounds. These vanadium compounds are considered as non-selective, competitive, reversible inhibitors of tyrosine phosphatases [16,21,28,58,59]. Some vanadium compounds, for example, peroxidovanadium complexes, are capable of oxidizing cysteine residues in the critically active site of phosphatases, which results in the irreversible inhibition of enzymatic activity [21]. In this way, vanadium exhibits a broad inhibitory capacity on the entire superfamily of tyrosine phosphatases, one that has been experimentally demonstrated using panels of various tyrosine phosphatases, such as PTP1B, SHP1, SHP2, TCPTP, PTP-MEG2, HePTP, HCPTPA, and HPTPb [58,60,61,62,63].

Despite initially being considered a non-selective inhibitor of tyrosine phosphatases, various studies have indicated the possibility of the more selective action of vanadium complexes. A review containing a compilation of results for the inhibition of different tyrosine phosphatases by several vanadium complexes indicated that they may exhibit varying degrees of inhibition [28]. In this compilation, some complexes showed 3 to 50 times stronger inhibition of PTP1B than other tyrosine phosphatases, while others showed no or little difference in inhibiting phosphatases other than PTP1B [60].

Most attention is paid to the importance of PTP1B phosphatase in diabetes and metabolic disturbances. However, ample evidence points to the important role of other tyrosine phosphatases in these disorders. Results suggest that SHP1 may be a potential target for muscle insulin resistance and insulin signaling during obesity, as well as the fact that it may be involved in the development of non-alcoholic fatty liver disease caused by diet-induced obesity [64,65,66]. Similarly, inhibition of SHP2 has been implicated in various signaling pathways, including those involved in potential anti-diabetic actions. The effect of inhibition decreased insulin resistance, decreased liver steatosis, enhanced insulin-induced suppression of hepatic glucose production, and impeded the development of insulin resistance after high-fat feeding [67,68,69]. Regarding LAR, the results indicate its involvement in the pathogenesis of metabolic disorders and suggest this phosphatase as a potential therapeutic target [70,71,72]. 

We have conducted studies on the inhibition of human recombinant PTP1B, the most extensively described biological target for vanadium activity, as well as on highly homologous human tyrosine phosphatases, such as LAR, SHP1, and SHP2, which may have potential relevance in metabolic diseases [27,29]. 

Screening tests and determination of IC50 confirmed that all ONS complexes are inhibitors of all tested phosphatases at a similar level to VOSO_4_ and BMOV. The rationale for employing these last two compounds as comparators of biological activity is grounded on their extensive research history and their established effectiveness in metabolic disorders. The absence of a comparator in most published studies poses challenges in comparing the relative activity of the studied vanadium compounds. Moreover, the published results of inhibition tests cover a wide range of results, for example, IC50 for PTP1B for VOSO_4_ ranges from 18 nM [73] to 380 nM [74]. This can be explained by differences in the substrate used (e.g., DiFMUP vs. p-NPP) or other analytical conditions. Our results for BMOV are consistent with previously published results of other authors (IC50 for PTP1B was 0.15 µM) [58]. Similarly, ONS complexes exhibited a comparable level of potency in inhibitory activity (IC50 was 0.14 and 0.11 µM for VC054 and VC059, respectively). The obtained results for the inhibition of other tested phosphatases align with the values reported in the literature, which commonly fall within the IC50 range of 0.1–0.9 µM [28]. 

The ONO complexes exhibited approximately 15–30 times weaker inhibition of PTP1B compared to BMOV and the ONS complexes. Similarly, the inhibition of the other tested tyrosine phosphatases was even several dozen times weaker for the ONO complexes. The comparators, BMOV and VOSO_4_, commonly used in previously published studies, exhibited a greater inhibitory effect on PTP1B than on SHP1 and SHP2. However, conflicting results have been reported by certain authors [75]. The differences in the activity of these comparators are small enough that VOSO_4_ and BMOV are reported to be non-selective for these tyrosine phosphatases. Referring to these data, our results for comparators as well as the studied complexes suggest that the differences in the tyrosine phosphatase inhibition strength may rather result from analytical conditions, such as different specific activity of the recombinant enzyme used for the analyses. Moreover, the presence of EDTA or a thiol-containing reducing agent (e.g., DDT), which modulates the catalytic activities of PTP1B and SHPs, may play a crucial role in modifying the complexes present in the reaction environment and determining the result [75].

The tested ONS and ONO complexes showed the inhibition of human non-tyrosine phosphatases: dual specificity phosphatase CDC25A and serine-threonine phosphatase PPA2. Very few publications on the biological activity of vanadium include the study of phosphatases from other classes and groups with different substrate specificity, such as CDC25A, which is the oncoprotein regulating the cell cycle [76,77,78]. Orthovanadate can inhibit the activity of calcineurin, a serine/threonine protein phosphatase that plays a crucial role in numerous signal transduction pathways [31,79]. Our results for PP2A are consistent with these findings and confirm that vanadium complexes inhibit not only protein tyrosine phosphatases but also serine/threonine phosphatases. Although few studies on the activity of vanadium compounds include these phosphatases, this direction seems important because over 98% of protein phosphorylation occurs on serine and threonine residues, and specific serine/threonine phosphatases are important and promising targets for drug development, particularly in diabetes treatment [33]. On the other hand, the non-specific effect of vanadium on phosphatases involved in cell cycle regulation may be a limitation of the clinical use of vanadium compounds in non-malignant diseases.

The ONS complexes showed higher activity in inhibiting human tyrosine phosphatases than the ONO complexes; however, it is interesting that their biological activity in cellular models of skeletal muscle and adipose tissue was lower than that of the ONO complexes. Similar results were observed, for example, for the ((CH_3_)_2_NO)_2_V(O)OH complex, which was not as good a PTP inhibitor as vanadate but was much more effective in inducing biological effects in cells by means of increasing glucose transport and glycogen synthesis [80].

We used the cell models of the main organs responsible for glucose metabolism, which are also target organs for diabetes pharmacotherapy. C2C12 myoblasts are a well-documented model with key characteristics of human muscle cells. This model is widely used in preclinical and pharmacological studies in the development of new drugs [81,82,83], including vanadium compounds [84,85,86]. Adipocytes of the 3T3-L1 line, also used in research on vanadium compounds, are similarly useful for the same reasons [87,88]. Impairment of insulin signaling and post-receptor intracellular mechanisms in insulin resistance is manifested by reduced glucose transport to myocytes and adipocytes, and its improvement is one of the main pharmacotherapeutic mechanisms in diabetes treatment [89,90]. In our study, we used radioactively labeled deoxy-D-glucose, a synthetic glucose analogue, which is transported to cells but does not undergo further metabolism in the glycolysis process. The product of the first stage of glycolysis inhibits this process non-competitively.

Different vanadium compounds, including its complexes, have an effect on basal and insulin-stimulated glucose uptake in adipocytes [91,92] as well as in myocytes [84,85,86]. Our ONS and ONO complexes also increased the transport of [14C]-deoxy-D-glucose into myocytes C2C12. Compared to the ONO complexes, the activity of the ONS complexes was lower, but despite this, they showed greater efficiency than VOSO_4_ and BMOV.

We extended the study of the transport of radiolabeled glucose analog to C2C12 myocytes with the study of glucose utilization (consumption) during a 24 h incubation with vanadium complexes. The study of glucose consumption gives a more complete picture of the pharmacological activity of the tested complexes because it depends on its consumption in cells, e.g., in the process of glycogenesis. In patients with type 2 diabetes, increased glycogen synthesis after administration of VOSO_4_ accounted for more than 80% of the increased glucose disposal in muscles [93]. In this case, the stimulation of glucose uptake by myocytes is independent of the effect of vanadium on the insulin receptor signal through the inhibition of tyrosine phosphatases [94]. We did not find any studies in which both glucose transport and glucose utilization were studied in the same cell model. 

As in the study of the transport of [14C]-deoxy-D-glucose in myocytes C2C12, the ONS complexes also showed a lower effect on glucose utilization than the ONO complexes. These effects were greater than those of VOSO_4_ and BMOV. In the experiment investigating glucose consumption by myocytes, no differences in activity were observed between the ONS and ONO complexes, in contrast to differences in the increase in [14C]-deoxy-D-glucose transport in the same cells.

Biotransformation of the complexes and vanadium speciation may be a possible cause. These processes can be critical to the observed activity and depend on the experimental condition and the time of incubation with the complexes, which in the study of the glucose analog transport to C2C12 myocytes was 6 h in total, and in the study of glucose consumption by these cells was 24 h. Biological activity depending on the incubation time has been demonstrated, among other things, in the case of vanadium complexes with 1,10-phenanthroline ligands. After 3 and 24 h, the cytotoxicity was different for the tested complexes, but after 72 h of incubation, all compounds showed equal activity. This supports the postulate that biological activity may depend more on the total concentration of vanadium than on the form in which it was used [95]. It was also shown that, regardless of the oxidation state in model vanadium (V), (IV), and (III) compounds, including vanadate and BMOV, after a 24 h incubation in the cell culture medium, approximately 75% of the total vanadium was vanadium (V). Similarly, vanadium speciation in HepG2 hepatocytes also varied with incubation time, with vanadium (IV) accounting for ≈20% to ≈70% of the total vanadium pool, largely independent of the vanadium complex used or the dominant vanadium oxidation state in the medium [96].

Prolonged incubation of the cells with vanadium complexes, as was the case during the study of the effect on glucose consumption, more closely corresponds to the actual conditions of clinical use, also allowing for the identification of possible cytotoxic effects. These effects were not observed, as cell viability was not lower than 80% of the vehicle control for all tested complexes. This level is interpreted as a lack of cytotoxic effect according to the ISO rules for in vitro cytotoxicity tests [56]. For some complexes, an increase in the signal in the viability assay was observed, which may have been due to the metabolic activation of the cells. A Resazurin-based reagent is reduced proportionally to the metabolic activity of cells as a result of the transference of electrons from NADPH+H^+^, the amount of which strictly depends on glucose metabolism [97]. Similar results for vanadium complexes have already been observed using C2C12 myocytes and the MTT test based on the same mechanism [98].

In this study, among the ONS complexes, only VC070 enhanced the transport of [14C]-deoxy-D-glucose in both models. The rest of complexes in this group did not demonstrate significant activity in 3T3-L1 adipocytes; however, their activity on myocytes was significant. This activity was higher than the activity of BMOV and VOSO_4_, although clearly lower than the activity of ONO complexes. 

VC070 differed from the other complexes of this group only in its starting amino acid, which was leucine. It can be assumed that this complex interacts with the amino acid transporter SLC7A5 (LAT1), which is the main leucine transporter in the body. It is also a transporter with broad substrate specificity, capable of transporting large hydrophobic neutral amino acids as well as many drugs [99,100]. Structural analogues of leucine and leucine-related compounds can also be transported by SLC7A5. The involvement of this transporter in the cellular transport of ruthenium complexes has also been suggested [101]. It is possible that in the body the donor complexes of ONS may be degraded and release the amino and carboxyl groups of the starting amino acids. On the other hand, other amino acids that were the starting amino acids in our ONS complexes can also be transported using this transporter. These complexes showed activity in myocytes where SLC7A5 has the highest expression and significantly lower activity in adipocytes where only VC070 with leucine as the starting amino acid was active. Differences in effect on adipocytes may be due to varying degrees of cellular amino acid uptake. The concentration of leucine in adipocytes is almost twice as high as, for example, phenylalanine or methionine, starting amino acids in other ONS complexes that did not show such a strong effect [102]. 

The possible synergistic effect of vanadium and the leucine derivative formed after the possible breakdown of the complex on the observed increase in glucose transport should also be considered. Leucine may enhance the transport of deoxy-D-glucose to myocytes by an insulin-independent mechanism as well as by the promotion of glucose transporter translocation to the plasma membrane [103,104]. 

The assumed involvement of the SLC7A5 transporter in the transport of ONS complexes to the cells could explain the lack of effect of ONS complexes on lipid accumulation in the model of non-alcoholic fatty liver disease. Oleic acid, used to induce hepatocyte steatosis, as a cis-unsaturated long-chain fatty acid, inhibits the transport of amino acids to cells [105,106]. The concentration of BSA in the medium (0.1%) may have been insufficient to bind free oleic acid and eliminate the effect on transport inhibition. This could explain why all of the tested ONS complexes showed high activity against the same hepatocytes in the gluconeogenesis inhibition experiment in which the cell medium did not contain oleic acid.

We undertook research on the hepatic effects of the ONS and ONO complexes because systemic metabolic disorders such as diabetes and obesity are closely related to the dysfunction of the liver as the central organ that maintains metabolic homeostasis. We conducted research in the model of non-alcoholic fatty liver disease (NAFLD), which is a continuum of liver dysfunction caused largely by dietary and lifestyle factors. Hepatocyte steatosis is often observed in insulin resistance and diabetes, and NAFLD itself, as it progresses, often leads to cirrhosis and hepatocellular carcinoma [107]. NAFLD is characterized by excessive storage of lipids in the cytosol of hepatocytes with impaired function, which is accompanied by a number of changes in the course of glucose and lipid metabolism and insulin resistance. These processes are of systemic importance in diabetes and obesity.

As mentioned above, the ONS complexes showed no reduction in lipid accumulation, while the two ONO complexes, VC067 and VC068, were found to be very active in inhibiting intracellular lipid accumulation. The lack of cytotoxic effects with reduction in cell number indicates that the observed effect was not due to the reduction in cell number. 

In the studies of other authors, similar effects were shown for vanadium (IV)-chlorodipicolinate in primary rat hepatocytes and hepatocytes HepG2 treated with palmitate. A significant decrease in the intracellular lipid contents in a dose-dependent manner ranging from 50 to 200 µM was observed. An analogous effect for this complex was also observed in liver tissue from mice fed a high-fat diet [108]. Most of the few studies on the effect of vanadium complexes on liver lipid disturbances used in vivo models and demonstrated effectiveness against the mechanisms responsible for fatty liver [109,110]. 

For some ONO complexes, we observed an increase in lipid accumulation in hepatocytes. Several possible causes must be considered in explaining this effect. This may indicate hepatotoxicity consistent with drug-induced steatohepatitis (DISH), which is a form of drug-induced liver injury (DILI) caused by various drugs [57]. In cell models, this can manifest as lipid accumulation without significant damage to cell membranes or cell death. The observed effects may be related to the presence of phenanthroline as a colligand, which may increase the cytotoxicity of vanadium complexes [111]. In the studies, the oxidation of V(IV) to V(V) was observed with the release of phenanthroline ligand, which in its free form was responsible for most of the observed cytotoxic effects [112]. Free phenanthroline is able to complex iron ions, facilitating their transport into the cell. Excessive iron pool in the cell may intensify the generation of a hydroxyl radical in the Fenton and Haber–Weiss reactions [113,114]. This may intensify oxidative stress, which is an important mechanism of hepatocyte steatosis. Vanadium compounds may have a pro-oxidative effect, depending on the degree of oxidation or the presence of other ligands, which may lead to lipid peroxidation in the liver [115,116]. 

Although metabolic processes aim to eliminate xenobiotics from the body as quickly as possible, they can transform non-toxic compounds into reactive metabolites at various stages. The structure of compounds can have sites or groups that are more susceptible to metabolic bioactivation, which can result in metabolites of varying chemical reactivity and thus varying degrees of toxicity [117]. 

Halogenated aromatic hydrocarbons, such as those studied ONO complexes with bromo moieties in the aromatic rings, undergo biodehalogenation. This process can generate particularly reactive intermediate metabolites. They can bind to nucleophilic sites in biological macromolecules, leading to their modification and cytotoxic and genotoxic effects [115,116,118]. In the case of the ONS complexes, the formation of chlorinated organic derivatives during their degradation is unlikely, as such structures are not components of the complexes and were not used in their synthesis.

The use of halogens in medicinal chemistry is becoming increasingly common due to their modulatory effects, and 14 out of the 50 molecules approved by the FDA in 2021 contain halogens [119]. The halogen substituent affects potency, inhibitory activity, or binding affinity, as well as having a pronounced effect on pharmacokinetic parameters, and it may determine cytotoxic effects. For example, in vitro cytotoxicity studies conducted on the human cancer cell line T47D have shown that the parent indole compound, which was essentially inactive, became the most active compound in the series when two hydrogen atoms on different rings were substituted with chlorine atoms. The modified compound exhibited over a 2500-fold improvement in cytotoxic potency [120]. 

Chloro-substitution is also employed in the synthesis of enhanced vanadium complexes, and the vanadium complexes with chloro-substituted Schiff base ligands exhibited increased hydrophobicity, hydrolytic stability, and ease of reduction compared to their non-chlorinated counterparts. These vanadium (V) complexes with substituted catecholates as co-ligands demonstrated potent in vitro anticancer activity [121]. On the other hand, the effects of the presence of halogens in the aromatic ring may not be unambiguous. Vanadium (IV)-chlorodipicolinate with a chlorine ligand at position 4 in the pyridine ring of dipicolinic acid showed beneficial effects in a mouse model of non-alcoholic fatty liver disease (NAFLD), effectively preventing hepatic steatosis and also mitigating oxidative stress and endoplasmic reticulum (ER) stress [109].

In our study, no certain and significant cytotoxic effects were observed. However, various reactions can occur in the metabolism of xenobiotics, and the intermediate and final metabolites can be difficult to predict. For our complexes, as with any drug candidate, the metabolic stability and toxicity of metabolites are important issues that should be considered in extended studies, especially in the later stages of preclinical development (hit-to-lead and lead optimization) [120].

The accumulation of lipids in hepatocytes observed under the influence of some ONO complexes may also result from the stimulation of glucose transport and the intensification of lipid synthesis in the hepatocytes.

All of the tested ONS and ONO complexes showed an effect on the gluconeogenesis process, inhibiting glucose synthesis in hepatocytes. Most of the studies on the effect of vanadium on gluconeogenesis come from earlier studies in animal models, and only a few studies deal with vanadium in the form of complexes [122,123,124,125]. All these studies confirm that the antidiabetic effect of vanadium is also manifested in the inhibition of hepatic gluconeogenesis, which is an important mechanism of glucose metabolism disorders and an effective therapeutic target [126]. Vanadium inhibits gluconeogenesis by inhibiting the expression of key genes that control this process: phosphoenolpyruvate carboxykinase (PEPCK) and glucose 6-phosphatase (G-6-Pase). This is done through the activation of AKT kinases with an enhanced phosphorylation of GSK-3 and FOXO, the main transcription factor of these genes [127].

Phosphorylation of AKT kinases has also been implicated as the primary mechanism of the antidiabetic action of vanadium compounds [128]. In this study, we demonstrated that both ONS and ONO complexes potentiate the phosphorylation of AKT in hepatocytes. This indicates that they act through the same shared mechanism, which is also the effector mechanism of insulin action. Abnormalities in the phosphorylation of AKT kinases are associated with the occurrence of insulin resistance in cells, and restoring their sensitivity to insulin is a key aspect of the pharmacotherapy of metabolic disorders. The insulin resistance of hepatocytes is caused, among other things, by exposure to increased concentrations of free fatty acids and hyperinsulinemia, and pro-inflammatory cytokines, such as TNF, intensify this process [129]. These factors were used to induce insulin resistance in the models used in this study.

The ONO complexes VC067 and VC068, which showed effects in both inhibiting lipid accumulation in the NAFLD model and inhibiting gluconeogenesis, can also reverse the effects of insulin resistance in hepatocytes. The few studies conducted in cellular models of hepatocytes have shown similar effects. Vanadium effectively reversed hepatocyte insulin resistance, induced by TNF [130], and the VO-OHpic vanadium complex’s mechanism of reversing insulin resistance was related to the inhibition of the dual specificity phosphatase, PTEN. Vanadium is an inhibitor of this phosphatase that regulates the activity of the AKT pathway [131].

Normal insulin signaling networks employ not only AKT but also extracellular-signal-regulated kinase (ERK). ERK has been implicated in the development of insulin resistance associated with obesity and type 2 diabetes mellitus. ERK is also the final effector of the pathway regulating the cell cycle and cell differentiation, and abnormal, uncontrolled activation is associated with neoplastic processes. In our study, all of the tested ONS and ONO complexes showed enhanced ERK phosphorylation. Dose- and time-dependent activation of the ERK signaling pathway through its phosphorylation was also found for vanadium compounds, which was associated with their anticancer effect. ERK activation through the PI3-K and ras pathway is also hypothesized to play an essential role in mediating the insulin-mimetic effects of vanadium compounds [22]. 

The observed differences in the effectiveness of ONS and ONO complexes in the different cell models and mechanisms may be due to the specific or selective effects of these complexes. Differences in the activity of vanadium complexes on various cell types have already been observed for bis-coordinated oxidovanadium (IV) complexes with the imidazolyl-carboxylate moiety which improved glucose uptake in cell cultures of myocytes C2C12, adipocytes 3T3-L1, and Chang cells [73]. For example, [VO(Im_2_COO)_2_] showed efficacy on 3T3-L1 adipocytes with no activity on C2C12 myocytes. [VO(Im_4_COO)_2_] showed the opposite effect and even decreased basal glucose utilization for 3T3-L1. Similar differences have also been observed for hepatocytes. Explaining the differences in the observed effects, we point to the potential possibility of structural rearrangement or decomposition of complexes, which were incubated with cells in two different media (DMEM or RPMI-1640, 10% FBS). On the other hand, two different cell models (myocytes C2C12 and Chang cells) were maintained in the same medium (RPMI-1640), yet differences in the effects of the complexes were observed. This indicates their selective action resulting from the structure of the complexes [73].

The selectivity of the effects of vanadium complexes against different cell lines cultured and incubated under the same experimental conditions has already been observed, among other things, for vanadium complexes with orotic and glutamic acids [132]. Similarly, structural modifications of the ligands of other complexes resulted in selectivity between cancer cell lines [101]. 

Among the possible reasons for the different intensity of the effects of vanadium complexes in different cell types is the interaction of the properties of the cells themselves and the physicochemical properties of the complexes. Preferential uptake of vanadium (V) complexes with hydrophobic organic ligands by cancer cells has been observed [133]. Such interactions may result from the different composition, structure, and properties of cell membranes in different tissues and specific changes in membranes in cancer cells [134]. Differences in these properties naturally also concern normal cells from various tissues. An example illustrating the influence of cell properties on interactions with vanadium complexes may be the degree of cell differentiation of 3T3-L1 adipocytes. The differentiation of these cells from preadipocytes to adipocytes is accompanied by changes in the amount and composition of intracellular lipids. 3T3-L1 mature adipocytes, as compared to preadipocytes of this cell line, showed a greater tendency to form vanadium IV, despite the predominance of form V in the medium [96]. Similarly, steatotic hepatocytes are characterized by altered biochemical processes and structural differences from normal hepatocytes, which include, among other things, modifications of cell membranes. This may determine the specific effects and activity of the ONS and ONO complexes.

## 4. Materials and Methods

### 4.1. Complex Synthesis and Characterization

#### 4.1.1. Materials and Analytical Methods

All reagents were of analytical grade (Sigma-Aldrich, Saint-Louis, MO, USA) and were used as supplied. Ethanol (98%) was of pharmaceutical grade, and all other solvents were of analytical grade and were used as supplied.

Microanalysis of carbon, hydrogen, nitrogen, and sulfur were performed using an Elementar Vario MICRO Cube elemental analyzer. The IR spectra for ligands L1–L5 were recorded using a Nicolet iSS FT-IR spectrophotometer Thermo Fisher Scientific, Waltham, MA, USA), while those for the complexes were recorded using a Bruker EQUINOX 55 FT-IR spectrophotometer in KBr pellets (Brucker, Billerica, MA, US). The electronic absorption spectra were recorded with Shimadzu UV-3600 UV-VIS-NIR spectrophotometer equipped with a CPS-240 temperature controller (Shimadzu Corp., Kyoto, Japan). The magnetic susceptibility measurements were performed on a MSB magnetic susceptibility balance (Sherwood Scientific, Cambridge, UK). NMR spectra were determined on a Bruker Avance II 300 MHz spectrometer (Brucker) (using TMS as an internal standard). 

#### 4.1.2. The Synthesis of Vanadium Complexes with ONS Schiff Base Ligands

##### Synthesis of 3-Hydroxythiocrotonic Acid Anilide

Stage 1 (Scheme 2 in Section 2): A solution of acetylacetone (acac) (40.1 g; 41.2 mL, 0.4 mol) in anhydrous DMF (150 mL) was cooled to 0 °C by immersing the reaction flask in ice water. Separately, sodium hydride NaH, without of paraffin oil, was prepared according to the following procedure: commercial NaH dispersed in paraffin oil (55–60%) was placed in a column with a glass filter, connected to a water pump, and washed with small portions of anhydrous diethyl ether (3 × 10 mL), thus removing the paraffin oil. Pure 100% NaH was dried by passing a stream of argon through the column, still connected to the water pump. Oil-free NaH (9.6 g; 0.4 mol) was added gradually to a cooled acac solution in DMF. During the addition of NaH, hydrogen was rapidly evolved, and then the contents of the flask solidified to a white mass, which was crushed with a baguette. After 1 h, phenyl isothiocyanate PhNCS (54.1 g; 51.2 mL; 0.4 mol) was added dropwise while the contents of the flask were stirred with a magnetic or mechanical stirrer. The reaction mixture turned a brown-red color. After one hour of stirring, the solution was poured onto 500 g of ice and acidified with HCl (1:1) to pH = 6. A brown oil precipitated, solidified over time, and turned yellow. It was a 2-acetyl derivative of 3-hydroxythiocrotonic acid anilide. 

Stage 2 (Scheme 2 in Section 2): A total of 3.41 g of sodium hydroxide and 65 mL of distilled water were introduced into a 250 mL beaker. The beaker was placed on a magnetic stirrer and its content was heated to 60 °C; then, 20 g of 2-acetyl-3-hydroxytiocrotonic acid anilide was dissolved in the contents of the beaker with vigorous stirring. The resulting solution was left in an ice bath for several hours. The resulting light-yellow precipitate of 3-hydroxythiocrotonic acid anilide was filtered off. The precipitate was crystallized from benzene, giving the product in the form of yellow flakes, m.p. 63–65 °C (lit. 64–66 °C). The yield after crystallization was 11.97 g (73%) of product III (Scheme 2). The product can also be crystallized from ethanol, but the crystallization efficiency was lower and did not exceed 55%.

##### Condensation Reaction of 3-Hydroxythiocrotonic Acid Anilide with an Amino Acid Salt 

The condensation reaction is illustrated in Scheme 3 in Section 2. 3-Hydroxythiocrotonic acid anilide (5.336 g; 27.61 mmol) was added to a 100 mL round bottom flask, which was dissolved in benzene (65 mL). Then, a small amount of DMF (1 mL) and the sodium salt of the amino acid (26.77 mmol) were triturated in a mortar. As amino acids, we used L-tryptophan, L-phenylalanine, L-leucine, L-methionine, and D/L-isoleucine. The flask was placed on a magnetic stirrer in an oil bath, and an azeotropic head and reflux condenser were attached. The reaction mixture was heated 4-14 hours to reflux; the oil bath temperature was 120 °C, until no more water separation was obtained. The formed yellowish precipitate IV (Scheme 3) was filtered after cooling, washed with toluene and then with petroleum ether and finally dried. In formulas of vanadium complexes with ONS ligands, the compound IV is marked as L_1_ to L_5_ (for R = L-tryptophan, L-phenylalanine, L-leucine, L-methionine, or D/L-isoleucine, respectively).

##### Syntheses of Complexes with ONS Ligands (VC054, VC059, VC070, VC073, VC109)

The synthesis of complexes with the ONS ligands is illustrated in Scheme 4 in Section 2. For a suspension of IV (3 mmol) in anhydrous THF (25 mL), 2 M HCl in diethyl ether (Et_2_O) (3 mmol; 1.5 mL) was added dropwise. The mixture was practically clarified during the addition of HCl in Et_2_O. Thirty minutes after the end of the addition of HCl in Et_2_O, VOCl_3_ (3 mmol; 0.52 g; 0.28 mL) was added dropwise. The color of the mixture changed from yellow to dark green during the addition of VOCl_3_. Then, 30 min after the end of the VOCl_3_ addition, the contents were centrifuged (4000 min^-1^, 4 min) and the supernatant was evaporated on a rotary evaporator (60 °C). The green-brown (olive) residue was dried in a vacuum oven (60 °C, 4 h). In Table 1 in Section 2, the formulas of ligand and complexes with the elemental analysis and IR spectra results are given.

#### 4.1.3. The Synthesis of Vanadium Complexes with ONO Schiff Base Ligands 

The syntheses of selected ONO complexes were previously described in [45,46,47]. 

##### Synthesis of ONO Complex [V(L_13_)(HL_13_)] (VC055) 

The 5-bromosalicylaldehyde (0.603 g, 3.0 mmol), 4-methoxybenzhydrazide (0.496 g, 3.0 mmol), and EtOH (20 mL) were refluxed under Ar for 15 min. Then, [V(acac)_3_] (0.528 g, 1.5 mmol) was added and reflux was continued for 20 min. Almost immediately, the formation of brown precipitation was observed, and the solution started to be light yellow. The mixture was cooled and filtered, and the complex was washed three times with excess of EtOH and dried in air. Yield: 0.936 g, 84%. MW = 746.27. Anal. Calcd. for C_30_H_23_Br_2_N_4_O_6_V: C, 48.28; H, 3.11; N, 7.51 %. Found: C, 48.03; H, 3.71; N, 6.94 %. The complex is paramagnetic, µ = 1.50 µ_B_. IR-ATR (cm^-1^): 3456 (w), 2956 (w), 1607 (s), 1509 (m), 1489 (w), 1456 (w), 1412 (w), 1365 (m), 1338 (w), 1308 (w), 1258 (m), 1177 (m), 1137 (w), 1087 (w), 1030 (m), 936 (w), 902 (w), 872 (w), 845 (w), 822 (w), 756 (w), 701 (w), 657 (w), 620 (w), 579 (w), 469 (w).

### 4.2. Methods of Biological Assays

#### 4.2.1. Materials

Molecular biology grade or pure for analysis reagents were used for biochemical assays. Media and consumables of appropriate quality and purpose used for cell cultures were obtained from the American Type Culture Collection (ATCC, LGC, Teddington, UK), Lonza (Basel, Switzerland), Thermo Fisher Scientific (Waltham, MA, USA), and Sigma-Aldrich (Saint-Louis, MO, USA).

#### 4.2.2. Inhibition of Human Recombinant Tyrosine Phosphatases

To determine the ability of the tested compounds for inhibition tyrosine phosphatases [46,135], human recombinant proteins were used (Sigma-Aldrich). The reactions were performed in black opaque 384-well microplates (PerkinElmer, Waltham, MA, USA). An equal volume of phosphatase solution in a reaction buffer (25 mM of 3-(N-morpholino)propanesulfonic acid (MOPS), 50 mM NaCl, 1 mM dithiothreitol (DTT), and 0.05% Tween-20 (pH 7.0)) was added to the solution of the tested compound on microplate. These solutions were dispensed using an automated injector. The following final concentrations of the tested phosphatases were used: PTP1B 50 ng/mL, SHP1 400 ng/mL, SHP2 50 ng/mL, LAR 5 ng/mL, and PTPRA 100 ng/mL. After 10 min, a solution of phosphate 6,8-difluoro-4-methyl (DiFMUP; Thermo Fisher Scientific) was added until its final concentration was 0.1 mM. Samples were incubated for 20 miN at 23 °C, and then the measurements of fluorescence intensity (excitation 355 and emission 560 nm) were performed on a multifunctional plate reader POLARstar Omega (BMG Labtech, Ortenberg, Germany). Assays were performed in triplicate. For the screening assays, the final concentration of the tested compounds was 1 µM. The results were expressed as percent inhibition of untreated control (enzyme with vehicle only) for the screening tests. For the determination of the half maximal inhibitory concentration (IC50), nine concentrations of the tested compound in the range 10 nM to 10 µM was assayed in triplicate. IC50 were calculated using GraphPad Prism version 6.0 software (GraphPad Software, Boston, MA, USA).

#### 4.2.3. Inhibition of Human Recombinant Non-Tyrosine Phosphatases 

For the inhibition assay of non-tyrosine phosphatases [136], human recombinant proteins were used (Sigma-Aldrich). The reactions were performed in black opaque 384-well microplates (PerkinElmer). To the solution of the tested complexes, an equal volume of a test solution of phosphatase was added. CDC25A were diluted in reaction buffer to the final concentration of 400 ng/mL as for tyrosine phosphatases (described in the section above), and PPA2 (final 0.5 U/mL) was diluted in 50 mM Tris-HCl, 0.05% Tween-20, and 125 µg/mL protease-free bovine serum albumin (pH 7.0). These solutions were dispensed using an automated injector. After 10 min, a solution of phosphate 6,8-difluoro-4-methyl (DiFMUP; Thermo Fisher Scientific) was added for the PPA2 sample until the final concentration 0.1 mM. For CDC25A samples, 3-O-methylfluorescein phosphate for the final concentration of 0.2 mM was added. Samples were incubated for 180 min at 23 °C, and then the measurements of fluorescence intensity (excitation 355 and emission 560 nm) were performed on a multifunctional plate reader POLARstar Omega (BMG Labtech). Assays were performed in triplicate. The results were expressed as percent of inhibition of untreated control (enzyme with vehicle solvent only).

#### 4.2.4. Cell Models and Culture Conditions

All cell lines were obtained directly from the ATCC (American Type Culture Collection). The passage number of cells used in the experiments was between 4 and 10. The evaluation of the functional stability of the cell lines was conducted based on the results for control compounds, which were tested in each experimental series and compared with the results obtained in the process of validation and optimization of experimental models. 

The myocyte C2C12 cell line (ATCC CRL-1772), a subclone of myoblasts from mouse muscles, was cultured according to standard protocol in DMEM supplemented with 10% fetal calf serum, 100 IU/ml penicillin, and 100 mg/ml streptomycin at 37 °C in 5% CO_2_. Cells were pleated on a 96-well microplate and after reach confluence were differentiated in medium with 2% horse serum. Differentiation medium was changed every 72 h. 

Adipocytes 3T3-L1 cell line (ATCC CRL-11605) derived from fibroblasts from mouse embryo tissue were cultured according to standard protocol in DMEM medium supplemented with 10% bovine calf serum, 100 IU/mL penicillin, and 100 mg/mL streptomycin at 37 °C in 5% CO_2_. Cells were seeded in 96-well poly-D-lysine-coated plates and cultured to reach confluency. The culture medium was then replaced for differentiation medium I (DMEM, 10% fetal bovine serum, 25 nM 3-isobutyl-1-methylxanthine (IBMX), 500 µM dexamethasone, and 670 nM/10 µg/mL human recombinant insulin). After 48 h of incubation (differentiation day 2), the medium was changed to differentiation medium II (DMEM, 10% fetal bovine serum, and 670 nM/10 µg/ml human recombinant insulin). 

The human hepatocyte HepG2 cell line (ATCC HB-8065) was cultured according to standard protocol in DMEM supplemented with 10% fetal bovine serum, 100 IU/mL penicillin, and 100 mg/mL streptomycin at 37 °C in 5% CO_2_. Cells were pleated on a 96-well microplate and were used at 18–24 h after seeding.

#### 4.2.5. Scintillation Proximity Assay for Uptake of Radiolabeled 2-deoxy-D-[U-14C]-glucose

Scintillation proximity assay (SPA) is a method to measure the real-time accumulation of radiolabeled substrates by adherent cells with no filtration needed. Radioactivity concentrated closer to the scintillator embedded in the plastic bottom of each well provides a stronger signal than the radiolabeled substrate in the culture medium [130]. Uptake of radiolabeled 2-deoxy-D-[U-14C]-glucose was conducted based on previous descriptions [137,138,139]. 

Hepatocytes HepG2, myocytes C2C12 (after 8 days differentiation), and adipocytes 3T3-L1 (after 11 days of differentiation) were cultured and maintained as described in the section above but pleated on a ScintiPlate TC 96-well microplate coated with a solid-phase scintillator (PerkinElmer). Hepatocytes and myocytes were washed, and culture medium was changed for medium with 0.5% BSA instead of serum. After 24 h incubation, the tested complexes were added. After 2 h incubation at 37 °C in 5% CO_2_, the medium was changed to low-glucose medium (1000 mg/L), and the tested complexes were added again, with further incubation for 4 h being conducted. Next, the cells were washed three times with Krebs–Ringer buffer (KRB) without glucose (1 mM MgSO_4_, 1 mM CaCl_2_, 136 mM NaCl, 4.7 mM KCl, and 10 mM HEPES; pH 7.4), and KRB with human recombinant insulin at a final concentration of 100 nM (Sigma-Aldrich, Saint-Louis, MO, USA) was added for 15 min. Wortmannin at a final concentration of 200 nM (Sigma-Aldrich ) was used as a negative control. After that, cells were washed three times with cold KRB, and cytochalasin B (10 mM final) was added for several wells as a ‘‘no uptake” control, which proceeded as shown below. 

Adipocytes were washed and the medium was changed to the basal medium. After 24 h incubation, the tested complexes were added. After 2 h incubation at 37 °C in 5% CO_2_, the medium was changed to DMEM medium without glucose, with 0.1% BSA free from fatty acid, 200 mM L-glutamine, and 100 mM pyruvate. The tested complexes were added again, and further incubation for 4 h was conducted. Next, cells were washed three times with Krebs–Ringer buffer (KRB) without glucose, and KRB with human recombinant insulin at a final concentration of 100 nM (Sigma-Aldrich) was added for 15 min. Wortmannin at a final concentration of 200 nM (Sigma-Aldrich) was used as the negative control. After that, cells were washed three times with cold KRB, and cytochalasin B (10 mM final) was added for several wells as “no uptake”.

For cells proceeded as above, 2-deoxy-D-[U-14C]-glucose (250–350 mCi/mmol, NEC720A, PerkinElmer) solution in KRB with a total activity of 0.03 mCi was added to each well. After 1 h incubation at 37 °C and 5% CO_2_, uptake was blocked by adding cytochalasin B (10 mM final). The radioactivity of samples was measured in a scintillation counter, MicroBeta Trilux 1450 (PerkinElmer). Non-specific radioactivity was subtracted from each result (cpm). Two independent experiments with triplicates were conducted. The final results were expressed as percent of the control that contained solvent only instead of the tested compound.

#### 4.2.6. Glucose Utilization in Myocytes

Differentiated myocytes C2C12 were used after 8 days of differentiation. The culture medium was changed for medium with 0.2% bovine serum albumin, 100 IU/mL penicillin, and 100 µg/mL streptomycin, and after 2 hours of incubation, the medium was changed to fresh medium containing the tested compounds. After 24 h incubation, supernatants were collected. 

Glucose concentration was determined based on enzymatic reaction with glucose oxidase and fluorometrically detection reaction end product using an AmplexRed Glucose/Glucose Oxidase Kit (Thermo Fisher Scientific) according to the manufacturer’s protocol. A total of 10 mL supernatant diluted 50× in 50 mM PBS (pH 7.4) and 10 mL reagents containing 4 U/mL glucose oxidase, 0.4 U/mL horseradish peroxidase, and 200 mM 10-acetyl-3,7-dihydroxyphenoxazine in 50 mM PBS (pH 7.4) was added to a black opaque 384-well OptiPlate and incubated for 30 min at 37 °C. All assays were conducted in triplicate. Fluorescence signal was measured at excitation of 530 nm and emission of 580 nm using a multimodal microplate reader POLARStar Omega (BMG Labtech), and glucose concentration in samples was calculated in MARS Data Analysis Software version 3.02.R2 (BMG Labtech) based on glucose standards. Glucose utilization was calculated as the differences between incubation medium without cells and medium with cells after incubation with the tested compound. The final results were expressed as the percent of controls containing cells and solvent only.

#### 4.2.7. Inhibition of Lipid Accumulation in the Cell Model of NAFLD

The induction of hepatic lipid accumulation and studies on the effect of vanadium complexes on this process were carried out based on previous descriptions [140,141,142]. Hepatocytes HepG2 in culture medium (as indicated above) were seeded on a collagen-I-coated 96-well microplate at an amount of 20,000 per well. After 16–24 h, the standard medium was changed for DMEM with 0.1% BSA fatty acid free and 0.5 mM oleic acid as sodium salt and tested compounds were added. Cells were incubated for 24 h, and the intracellular lipid content was evaluated using AdipoRed Adipogenesis Assay Reagent (Lonza) according to the manufacturer’s protocol. Hepatocytes were washed with PBS with calcium and magnesium, and 5 µL AdipoRed Reagent in 200 µL PBS was added, with the mixture incubated for 10 min at 22 °C. Fluorescence signal proportional to lipid content in cells was measured at excitation in 530 nm and emission in 550 nm using a multimodal microplate reader POLARStar Omega (BMG Labtech). The results were normalized to the untreated control (cells with solvent only), wherein the intensity of fluorescence was taken as 100%.

#### 4.2.8. Inhibition of Hepatic Gluconeogenesis

Experiments on the inhibition of hepatic gluconeogenesis in HepG2 hepatocytes by the vanadium complexes were carried out based on a previous description with some modifications [143]. Hepatocytes HepG2 in culture medium (as indicated above) were seeded on a half-area 96 microplate at the amount of 50,000 per well. After 16–24 h, cells were washed with PBS three times and one time with experimental medium (DMEM without glucose with 0.1% BSA, 2 µM L-glutamine, 15 mM HEPES, 1 mM sodium pyruvate, 10 mM calcium lactate, 100 IU/mL penicillin, and 100 mg/mL streptomycin). Cells were incubated in this medium for 3 h at 37 °C in 5% CO_2_, and the next medium was changed to the experimental medium with additional gluconeogenesis inductors: 10 µM dexamethasone, 100 µM 8-bromoadenosine 3′,5′-cyclic monophosphate sodium salt (8-Br-cAMP), and 500 µM 3-isobutyl-1-methylxanthine (IBMX). After adding the test compounds, the cells were incubated for 3 h at 37 °C in a 5% CO_2_ microplate, and then the supernatant was collected for glucose assay (as described in Section 4.2.6). Glucose synthesis was calculated as glucose concentration differences between experimental medium without cells and medium with cells after incubation with the tested compound. The final results were expressed as the percent of inhibition of glucose synthesis by cells incubated with solvent only.

#### 4.2.9. Hyperinsulinemia Condition and Induction of Insulin-Resistant Hepatocytes 

The induction of insulin-resistant hepatocytes HepG2 were carried out based on previous descriptions [144,145]. Hepatocytes HepG2 in culture medium (as described in the section above) were pleated on a ScintiPlate TC 96-well microplate coated with a solid phase scintillator (PerkinElmer). After 16–24 h, the medium was changed to a medium with the addition of human recombinant insulin at a concentration of 10 µg/mL or with insulin-resistant inductors: 10 ng/mL TNF-α or oleic acid as sodium salt, and cells were incubated for the next 48 h at 37 °C in 5% CO_2_. Next, the compounds in fresh medium were added, further incubation was carried out for 24 h, and the scintillation proximity assay for the uptake of radiolabeled 2-deoxy-D-[U-14C]-glucose was conducted as described in the section above.

#### 4.2.10. Cytotoxicity Assay (Cell Membrane Damage)

The bioluminescent ToxiLight assay (Lonza) was used as a highly sensitive cytotoxicity assay designed to measure cell membrane damage [146]. After incubation of cells with the tested compounds, 10 μL of cell supernatant was deposited in a new 96-well plate. Then, 40 μL of the Adenylate Kinase Detection Reagent (AKDR) was added per well. After 5 min of incubation at 22 °C, the luminescence intensity was measured in a multifunction plate reader (POLARstar Omega, BMG Labtech). The results were expressed as percent of the signal of the untreated control (cells with solvent only).

#### 4.2.11. Homogeneous Proximity-Based Assay for AKT and MAPK/ERK Phosphorylation 

Hepatocytes HepG2 were seeded on a 96-well microplate and incubated in culture medium without serum for 3 h and were then washed three times with Krebs–Ringer buffer (KRB) (115 mM NaCl, 4.7 mM KCl, 1.28 mM CaCl_2_, 1.2 mM MgSO_4_, 1.2 mM KH_2_PO_4_, 10 mM NaHCO_3_, 25 mM HEPES (pH 7.4)). The tested compounds were added in serum-free culture medium, and cells were incubated for 30 min for AKT and 20 min for ERK assay at 37 °C in 5% CO_2_ medium. Next, cells were washed, incubated for a further 1 and 3 h, and washed again before the assay. 

Protein phosphorylation was assayed using a homogenous proximity-assay [147] with an AlphaScreen SureFire Akt 1/2/3 (p-Ser473) Assay Kit and an AlphaScreen SureFire ERK 1/2 (p-Thr202/Tyr204) Assay Kit (PerkinElmer) according to the manufacturer’s manual. Cells were lysed with Lysis Buffer with shaking for 10 min (350 rpm). A total of 30 μL of the lysate was transferred to the white opaque 96 well half-area assay plate, and 10 μL Acceptor Mix was added. The microplate was agitated on a plate shaker for 2 min (350 rpm) and then incubated for 2 h at 23 °C. A total of 10 μL of Donor Mix was added to the wells under subdued light, and the microplate was agitated on a plate shaker for 2 min (350 rpm) and then incubated for 1 h (Akt) or 3 h (ERK) at 23 °C in darkness. The alpha signal was measured in standard mode using a multimodal microplate reader POLARStar Omega (BMG Labtech). The results were expressed as a ratio of phosphorylated protein to total protein and normalized to the untreated control (cells with solvent only), wherein the intensity of the signal was taken as 100%.

### 4.3. Statistical Methods

Unless otherwise indicated in the text, statistical analysis of the results was performed using analysis of variance followed by the Dunnett test for post hoc comparisons with *p* < 0.05. All tests were performed using GraphPad Prism version 6.0 for Windows, (GraphPad Software, Boston, MA, USA).

## 5. Conclusions

In this study, we confirmed that the novel synthetized and characterized ONS-type vanadium (V) complexes with thioanilide derivatives of amino acids showed pharmacological activity in the cell models of metabolic disturbances.

Our research approach with a wide range of models and investigated mechanisms, adequate for the wide range of activity of vanadium complexes, allowed for the identification of complexes that specifically act on myocytes, adipocytes, and hepatocytes as well as the pathogenetic processes of metabolic disorders, including NAFLD.

The ONS complexes showed the inhibition of human protein tyrosine phosphatases (PTP1B, LAR, SHP1, and SHP2) in the submicromolar range (IC50 in the range of 13–141 nM), as well as the inhibition of non-tyrosine phosphatases (CDC25A and PPA2), similar to bis(maltolato)oxidovanadium(IV)(BMOV). The ONO complexes showed weaker inhibition than ONS complexes (IC50 in the range 0.23–4.26 µM).

The simultaneous testing of ONO-type complexes made possible a direct comparison of the potential and strength of action between the two groups of complexes. In addition to the differences in the pharmacological effects of the ONS and ONO group complexes, within each of these groups, we identified complexes with effects that distinguished them from other complexes in the same group. VC070 was the only ONS type complex that enhanced the transport of [14C]-deoxy-D-glucose in C2C12 myocytes and 3T3-L1 adipocytes (158 ± 10% and 159 ± 14% of the control, respectively). In contrast, the ONO-type complex VC032 caused an increase in this transport into myocytes (266 ± 36% of control) and no effect in adipocytes (97 ± 14%).

VC068, from the ONO group complexes, showed high activity in all of the employed cell models. For example, this complex at a concentration of 50 µM increased the transport of [14C]-deoxy-D-glucose in C2C12 myocytes (218 ± 10%) and 3T3-L1 adipocytes (177 ± 3% of the control). Moreover, VC068 inhibited gluconeogenesis in hepatocytes HepG2 by 79 ± 18% and decreased lipid accumulation in the non-alcoholic fatty liver disease model using the same cells by 36% ± 7% (relative to controls).

Our study serves as the basis for further research on the selective or specific effects of vanadium complexes, which may help develop vanadium complexes with improved pharmacological properties.

## Figures and Tables

**Table 1:** The formulas of the ONS complexes with the elemental analyses and IR spectra results.

Compound	Starting Amino Acid	Structural Formula of the Complex	Elemental Analysis of the Complex * [%]	IR Bands [cm^−1^]
**VC054**	L-tryptophan	[VOCl_2_(L_1_)]·1.5Et_2_O ·6.5HCl 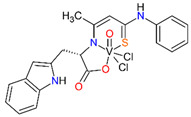	C, 37.38; **37.56** H, 4.86; **4.73** N, 5.22; **4.87** S, 3.91; **3.71**	3192 (w), 3054 (w), 2922 (s), 1709 (w), 1645 (s), 1619 (w), 1523 (w), 1491 (w), 1430 (m), 1396 (w), 1370 (m), 1253 (w), 1199 (w), 1120 (w), 1040 (w), 983 (m), 807 (w), 744 (m), 701 (m), 599 (w)
**VC059**	L-phenylalanine	[VOCl_2_(L_2_)]·0.5Et_2_O·1.5HCl 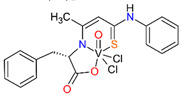	C, 44.62; **44.40** H, 4.35; **4.36** N, 4.58; **4.93** S, 5.55; **5.65**	3059 (s), 1709 (w), 1624 (s), 1606 (s), 1524 (w), 1497 (s), 1439 (m), 1407 (w), 1349 (w), 1221 (w), 1131 (w), 1078 (w), 1025 (w), 986 (m), 813 (w), 755 (m), 701 (m), 600 (w)
**VC070**	L-leucine	[VOCl_2_(L_3_)]·0.5Et_2_O·3HCl 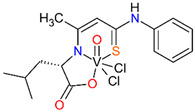	C, 36.54; **36.72** H, 4.66; **4.79** N, 4.88; **4.76** S, 5.40; **5.45**	3070 (s), 2958 (s), 1714 (w), 1618 (s), 1560 (w), 1523 (m), 1497 (s), 1447 (m), 1412 (w), 1231 (w), 1179 (w), 1120 (w), 1078 (w), 988 (m), 946 (w), 876 (w), 760 (m), 696 (m), 603 (w)
**VC073**	L-methionine	[VOCl_2_(L_4_)]·1.5THF·3HCl 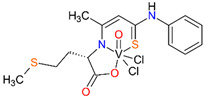	C, 37.42; **37.21** H, 4.81; **4.91** N, 4.05; **4.13** S, 9.85; **9.46**	3181 (w), 3001 (s), 2921 (s), 1709 (w), 1614 (s), 1603 (s), 1560 (w), 1528 (s), 1491 (s), 1433 (s), 1346 (w), 1243 (w), 1136 (w), 1078 (w), 983 (m), 760 (m), 692 (m), 596 (w)
**VC109**	D/L-isoleucine	[VOCl_2_(L_5_)]·3HCl 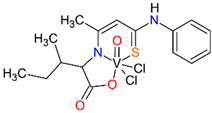	C, 34.77; **34.84** H, 4.38; **4.20** N, 5.07; **5.08** S, 5.80; **5.81**	3338 (w), 3059 (w), 2970 (s), 2939 (w), 2885 (w), 1729 (w), 1606 (s), 1520 (s), 1489 (s), 1447 (m), 1389 (w), 1350 (w), 1218 (w), 1180 (w), 1114 (w), 986 (m), 865 (w), 758 (m), 696 (m), 599 (w)

* The calculated elemental analyses are in bold.

**Table 2:** The formulas of the ONO complexes and the hydrazide components of Schiff base ligands (L_n_) used in synthesis with the reference for previously described complexes; phen = 1,10-phenanthroline.

Compound	Formula	L_n_ Components (1:1 Molar Ratio)		Ref.
		Aldehyde	Hydrazide	
**VC013**	[VO(L_6_)(phen)]⋅H_2_O	5-bromosalicyl-aldehyde	2-hydroxybenzhydrazide	[45]
**VC029**	[V(L_7_)(HL_7_)]		2-hydroxybenzhydrazide	[46]
**VC032**	[V(L_8_)(HL_8_)]⋅H_2_O		benzhydrazide	[47]
**VC046**	[VO(L_9_)(phen)]⋅2H_2_O		4-hydroxybenzhydrazide	[46]
**VC048**	[VO(L_10_)(phen)]⋅0.5H_2_O		4-tertbutylbenzhydrazide	[47]
**VC055**	[V(L_11_)(HL_11_)]		4-methoxybenzhydrazide	- *
**VC050**	[VO(L_11_)(phen)]		4-chlorobenzhydrazide	[46]
**VC067**	[V(L_12_)(HL_12_)]		3-hydroxy-2-naphthoic acid hydrazide	[46]
**VC068**	[V(L_13_)(HL_13_)]		4-nitrobenzhydrazide	[46]

* Described in this study.

**Table 3:** Inhibition of human tyrosine phosphatases by the ONS and ONO vanadium complexes at the concentration of 1 µM. Results are expressed as percentage of inhibition of the control (solvent only). Standard deviation did not exceed 6%.

		PTP1B	LAR	SHP1	SHP2
Non-vanadium control	Suramin	7	9	17	10
	(NH_4_)_6_Mo_7_O_24_	33	28	40	47
Vanadium comparators	VOSO_4_	62	44	70	77
	BMOV	77	58	76	82
ONS complexes	VC054	78	67	84	87
	VC059	79	71	82	87
	VC070	79	69	84	88
	VC073	74	63	82	86
	VC109	70	56	80	83
ONO complexes	VC013	60	66	67	74
	VC029	31	25	62	68
	VC032	9	8	44	48
	VC046	34	37	62	66
	VC048	36	35	62	64
	VC050	25	19	53	57
	VC055	24	18	56	57
	VC067	42	39	70	74
	VC068	40	33	70	74

The backgrounds of the table cells represent a two-color scale heat map. 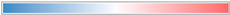 The darkest red would indicate 100% inhibition; the darkest blue would indicate 0%; and the middle of the scale, corresponding to 50% inhibition, is colorless.

**Table 4:** Inhibition of the human tyrosine phosphatases by the selected ONS and ONO complexes.

		IC50 [nM]				Log IC50 ± SD			
		PTP1B	LAR	SHP1	SHP2	PTP1B	LAR	SHP1	SHP2
Non-vanadium control	(NH_4_)_6_Mo_7_O_24_	58	38	26	26	−7.24 ± 0.06	−7.42 ± 0.04	−7.59 ± 0.13	−7.58 ± 0.04
Vanadium comparators	VOSO_4_	99	95	17	13	−7.00 ± 0.07	−7.88 ± 0.10	−7.77 ± 0.02	−7.77 ± 0.02
	BMOV	149	140	14	8	−6.83 ± 0.02	−8.09 ± 0.11	−7.86 ± 0.03	−7.86 ± 0.03
ONS complexes	VC054	141	112	20	20	−6.85 ± 0.01	−7.70 ± 0.06	−7.56 ± 0.02	−7.56 ± 0.02
	VC059	107	76	26	13	−6.97 ± 0.01	−7.87 ± 0.06	−7.59 ± 0.04	−7.59 ± 0.04
ONO complexes	VC050	4263	1714	657	273	−5.37 ± 0.03	−6.56 ± 0.04	−6.18 ± 0.03	−6.18 ± 0.03
	VC068	2034	619	517	235	−5.69 ± 0.02	−6.63 ± 0.03	−6.29 ± 0.02	−6.29 ± 0.02

**Table 5:** Inhibition of human non-tyrosine phosphatases and PTPRA by the selected ONS and ONO complexes. Results are expressed as percentage inhibition of control (solvent only) ± SD.

		CDC25A		PP2A	
		10 µM	1 µM	10 µM	1 µM
Non-vanadium comparator	(NH_4_)_6_Mo_7_O_24_	51 ± 1	34 ± 1	53 ± 3	18 ± 2
Vanadium comparators	VOSO_4_	62 ± 1	54 ± 2	40 ± 3	32 ± 2
	BMOV	61 ± 1	54 ± 1	63 ± 4	52 ± 15
ONS complexes	VC054	65 ± 3	51 ± 2	75 ± 7	41 ± 15
	VC059	68 ± 3	55 ± 3	54 ± 9	44 ± 1
ONO complexes	VC050	38 ± 1	12 ± 8	51 ± 4	24 ± 3
	VC068	62 ± 2	54 ± 3	55 ± 15	26 ± 12

## Data Availability

Data is contained within the article.

## Supplementary Materials

The following supporting information can be downloaded at https://www.mdpi.com/article/10.3390/ph17020229/s1. Figure S1: ^1^H-NMR spectrum of L_1_. Figure S2: ^13^C-NMR spectrum of L_1_. Figure S3: ^1^H-NMR spectrum of L_2_. Figure S4: ^13^C-NMR spectrum of L_2_. Figure S5: ^1^H-NMR spectrum of L_3_. Figure S6: ^13^C-NMR spectrum of L_3_. Figure S7: ^1^H-NMR spectrum of L_4_. Figure S8: ^13^C-NMR spectrum of L_4_. Figure S9: ^1^H-NMR spectrum of L_5_. Figure S10: ^13^C-NMR spectrum of L_5_. Figure S11: IR-ATR spectrum of L_1_. Figure S12: IR-ATR spectrum of L_2._ Figure S13: IR-ATR spectrum of L_3._ Figure S14: IR-ATR spectrum of L_4_. Figure S15: IR-ATR spectrum of L_5_. Figure S16: IR-ATR spectrum of VC054. Figure S17: IR-ATR spectrum of VC059. Figure S18: IR-ATR spectrum of VC070. Figure S19: IR-ATR spectrum of VC073. Figure S20: IR-ATR spectrum of VC109. Figure S21: IR-ATR spectrum of VC055. Figure S22: UV–VIS spectra of complex VC054 in DMSO-H_2_O mixture. Figure S23: UV–VIS spectra of complex VC059 in DMSO-H_2_O mixture. Figure S24: UV–VIS spectra of complex VC070 in DMSO-H_2_O mixture. Figure S25: UV–VIS spectra of complex VC073 in DMSO-H_2_O mixture. Figure S26: UV–VIS spectra of complex VC055 in DMSO-H_2_O mixture. Figure S27: Qualitative UV–VIS spectra of complex VC054 in different solvents. Figure S28: Qualitative UV–VIS spectra of complex VC059 in different solvents. Figure S29: Qualitative UV–VIS spectra of complex VC070 in different solvents. Figure S30: Qualitative UV–VIS spectra of complex VC073 in different solvents. Figure S31: Qualitative UV–VIS spectra of complex VC109 in different solvents. Figure S32: Qualitative UV–VIS spectra of complex VC055 in different solvents.
