# Supplementary material for: Vanadium Complexes with Thioanilide Derivatives of Amino Acids: Inhibition of Human Phosphatases and Specificity in Various Cell Models of Metabolic Disturbances
Source: Pharmaceuticals (Basel). 2024 Feb 9;17(2):229. doi: 10.3390/ph17020229 (PMC10892041; doi:10.3390/ph17020229)
Supplement: Supplementary file 1 [file pharmaceuticals-17-00229-s001.zip › pharmaceuticals-2812494-supplementary.pdf]

Supplementary

Vanadium Complexes with Thioanilide Derivatives of Amino Acids: Inhibition of Human Phosphatases and Specificity in Various Cell Models of Metabolic Disturbances

Grzegorz Kazek [1]*, Monika Głuch-Lutwin [2], Barbara Mordyl [2], Elżbieta Menaszek [3], Monika Kubacka [4], Anna Jurowska [5], Dariusz Cież [6], Bartosz Trzewik [6], Janusz Szklarzewicz [5] and Monika Papież [3]

[1] Department of Pharmacological Screening, Chair of Pharmacodynamics, Faculty of Pharmacy, Jagiellonian University Medical College, Medyczna 9, 30-688 Krakow, Poland; grzegorz.kazek@uj.edu.pl

[2] Department of Radioligands, Chair of Pharmacobiology, Faculty of Pharmacy, Jagiellonian University Medical College, Medyczna 9, 30-688 Krakow, Poland; monika.gluch-lutwin@uj.edu.pl; barbara.mordyl@uj.edu.pl

[3] Department of Cytobiology, Chair of Pharmacobiology, Faculty of Pharmacy, Jagiellonian University Medical College, Medyczna 9, 30-688 Krakow, Poland; elzbieta.menaszek@uj.edu.pl; monika.papiez@uj.edu.pl

[4] Chair of Pharmacodynamics, Faculty of Pharmacy, Jagiellonian University Medical College, Medyczna 9, 30-688 Krakow, Poland; monika.kubacka@uj.edu.pl

[5] Coordination Chemistry Group, Faculty of Chemistry, Jagiellonian University, Gronostajowa 2, 30-387, Krakow, Poland; jurowska@chemia.uj.edu.pl; szklarze@chemia.uj.edu.pl

[6] Department of Organic Chemistry, Faculty of Chemistry, Jagiellonian University, Gronostajowa 2, 30-387, Krakow, Poland; ciez@chemia.uj.edu.pl; trzewik@chemia.uj.edu.pl

*Correspondence: grzegorz.kazek@uj.edu.pl

^{1}H-NMR and ^{13}C-NMR spectra of the L_1-L_5 ligands.

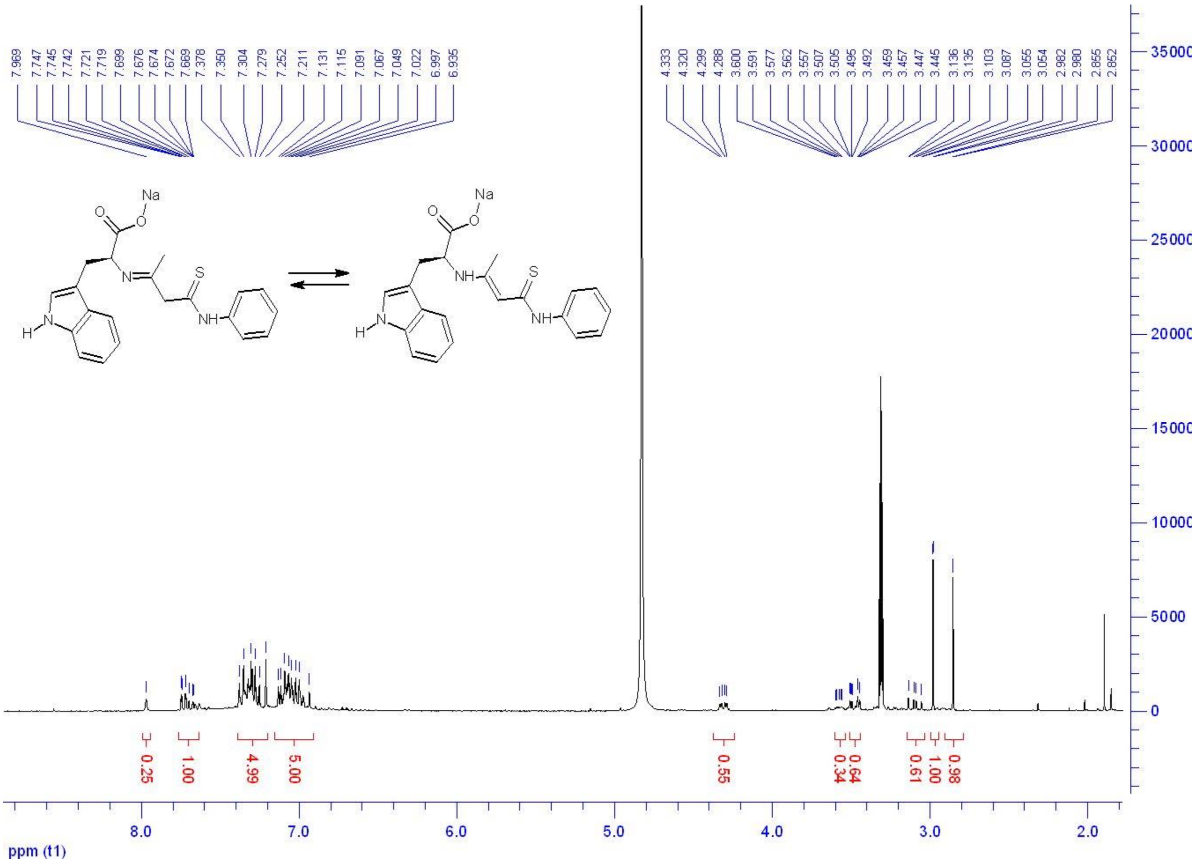

Figure S1. ^{1}H-NMR spectrum of L_1. (CD_3OD). σ [ppm] = 7.96 (br. s, NH), 7.74 (m, 1H, Ar-H), 7.72 (m, 1H, Ar-H), 7.28 (m, 4H, Ar-H), 7.21 (br. s, NH), 7.05 (m, 5H, Ar-H), 4.31 (dd, 1H, J_{HH} = 3.6 and 9.9 Hz, CH imine), 3.58 (m, 1H, CH enamine), 3.47 (dd, 2H, J_{HH} = 3.7 and 15.0 Hz, CH_aH_b enamine), 2.94 (dd, 1H, J_{HH} = 10.0 and 15.0 Hz, CH_aH_b imine), 2.99 (s, 3H, CH_3 enamine), 2.85 (s, 3H, CH_3 imine).

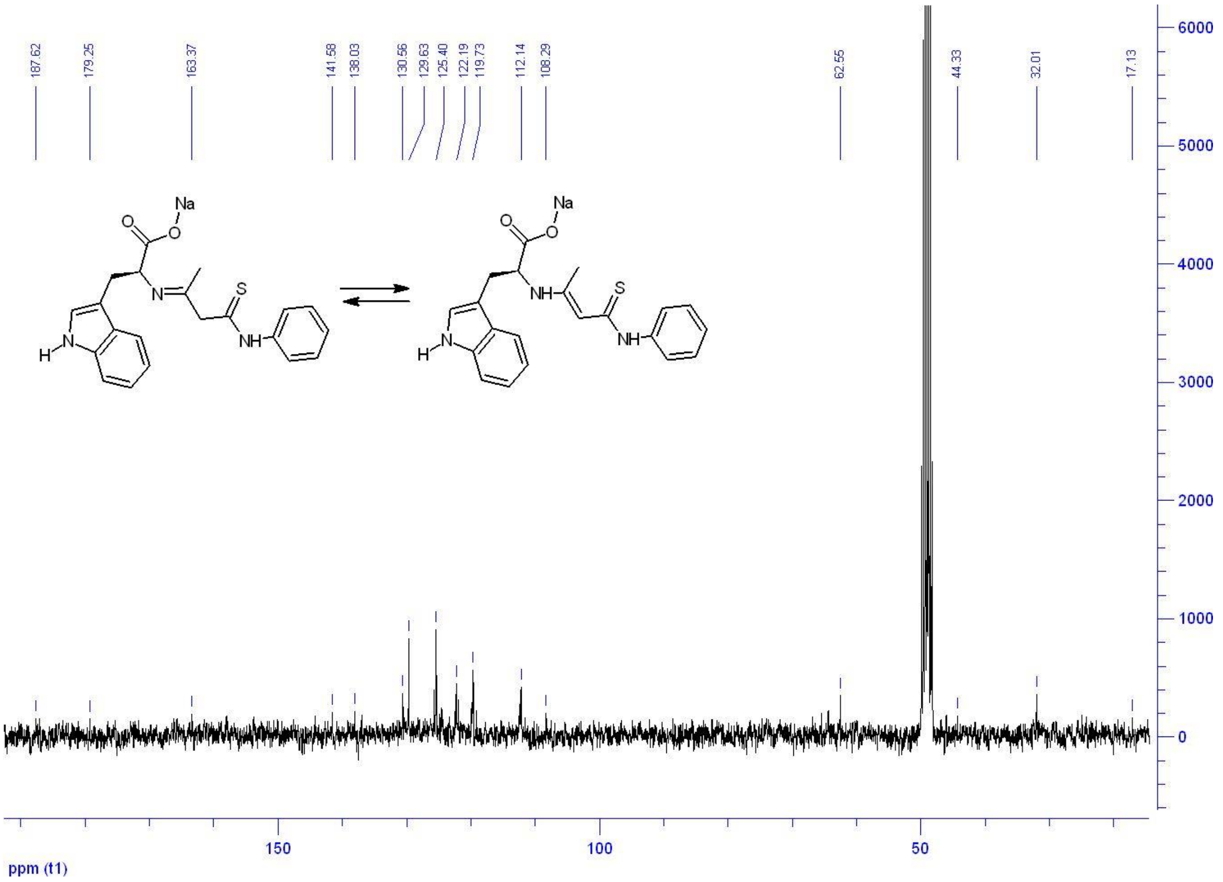

Figure S2. ^{13}C-NMR spectrum of L1. (CD_3OD)σ [ppm] = 187.6 (C=S), 179.2 (COONa), 163.4 (C=N), 141.6 (Ar), 138.0 (Ar), 130.5 (Ar), 129.6 (Ar), 125.4 (Ar), 122.2 (Ar), 119.7 (Ar), 112.1 (Ar), 108.3 (Ar), 62.5 (CH), 44.3 (CH_2), 32.0 (CH_2), 17.1 (CH_3).

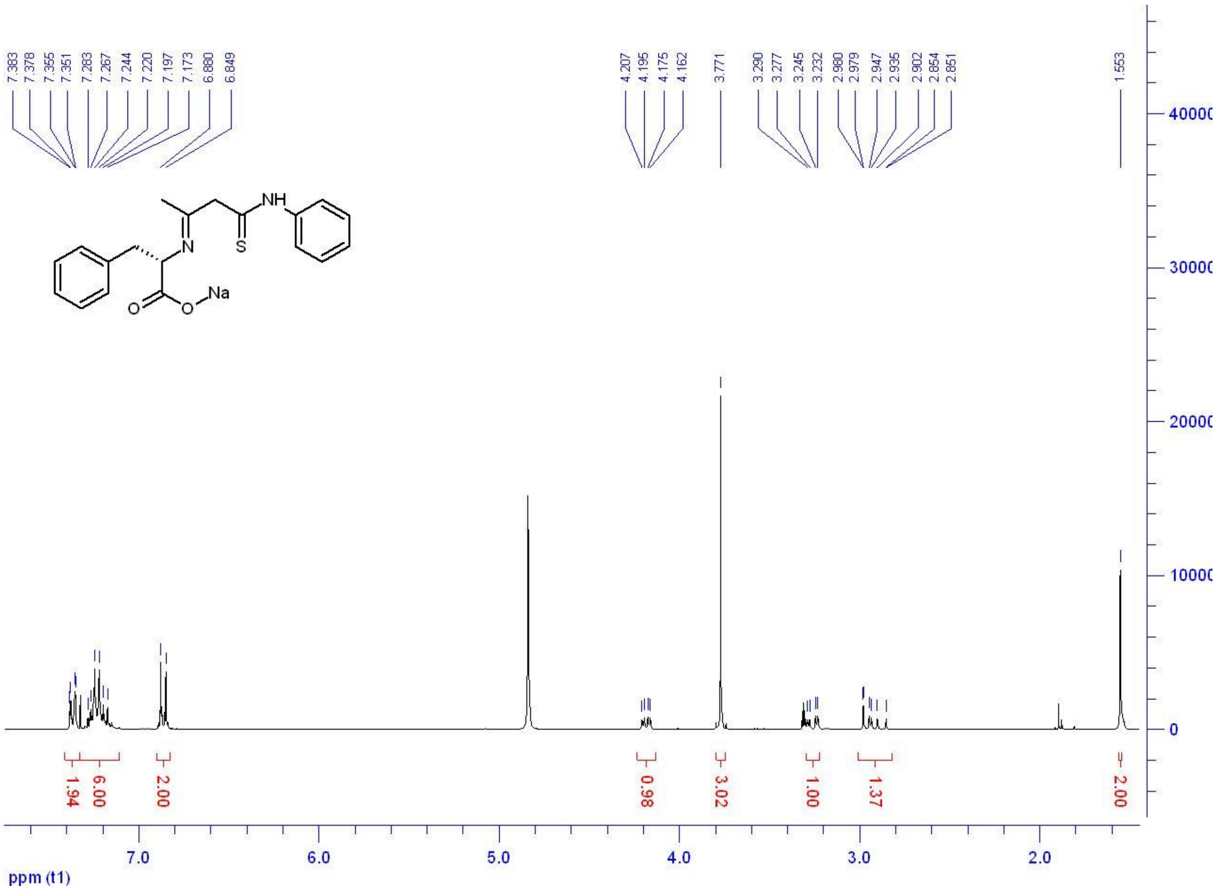

Figure S3. ^{1}H-NMR spectrum of L_2. (CD_3OD). σ [ppm] = 7.37 (d, 2H, J_{HH} = 6.7 Hz, Ph-H), 7.23 (m, 6H, Ph-H), 6.86 (d, 2H, J_{HH} = 9.1 Hz, Ph-H), 4.18 (dd, 1H, J_{HH} = 3.8 and 9.7 Hz, CH), 3.77 (s, 3H, CH_3), 3.26 (dd, 1H, J_{HH} = 3.8 and 13.8 Hz, CH_aH_b), 2.94 (dd, 1H, J_{HH} = 9.8 and 13.8 Hz, CH_aH_b), 1.55 (s, 2H, CH_2).

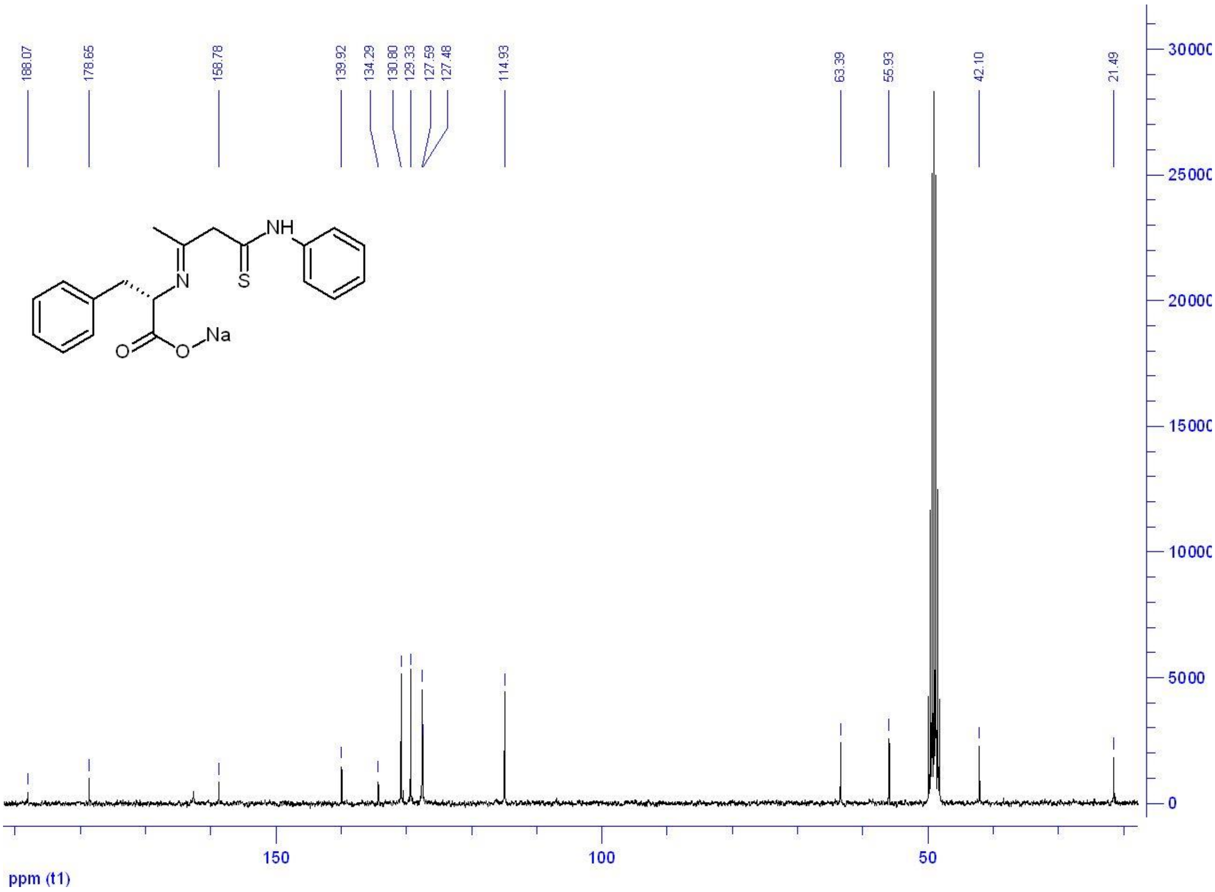

Figure S4. ^{13}C-NMR spectrum of L_2. (CD_3OD)σ [ppm] = 188.1 (C=S), 178.6 (COONa), 158.8 (C=N), 139.9 (Ph), 134.3 (Ph), 130.8 (Ph), 129.3 (Ph), 127.6 (Ph), 127.5 (Ph), 114.9 (Ph), 63.4 (CH), 55.9 (CH_2), 42.1 (CH_2), 21.5 (CH_3).

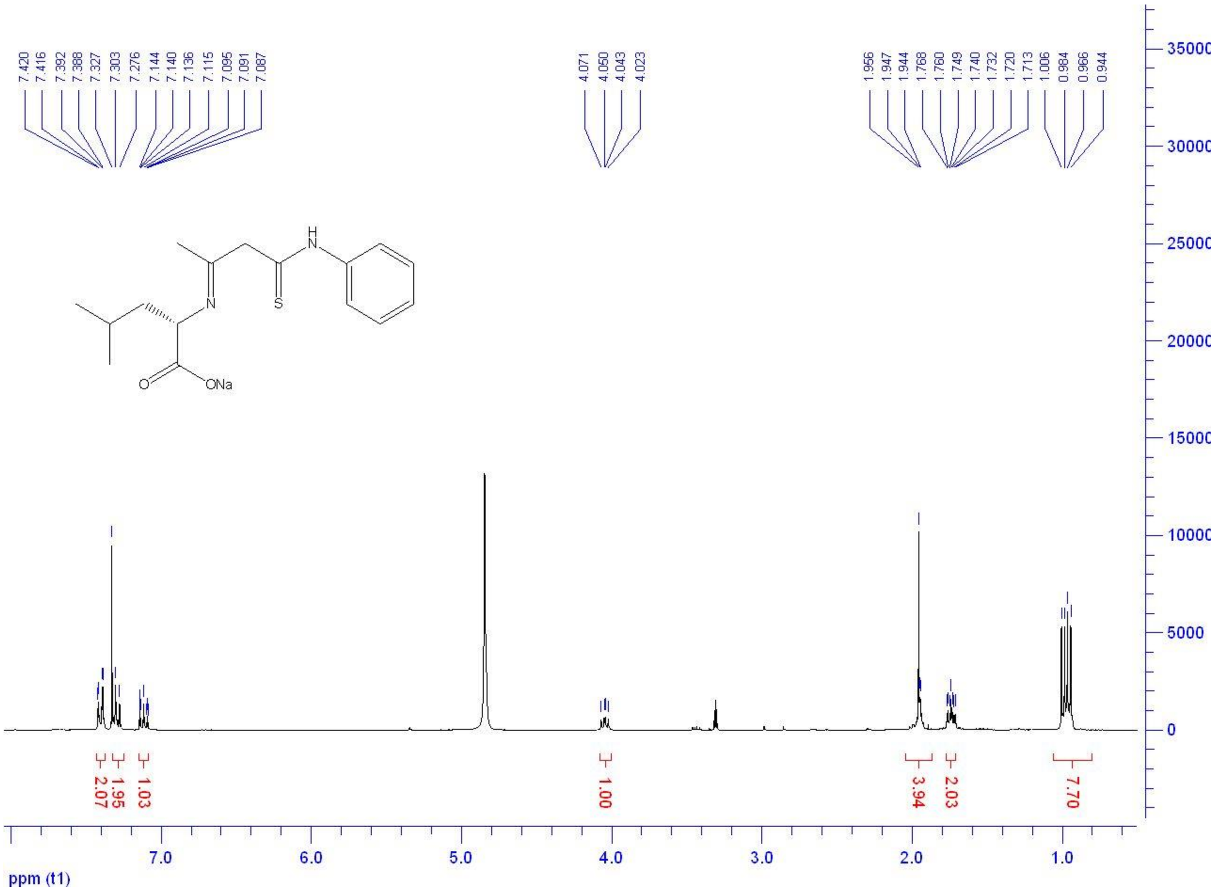

Figure S5. ^{1}H-NMR spectrum of L_3. (CD_3OD). σ [ppm] = 7.40 (d, 2H, J_{HH} = 8.5 Hz, Ph-H), 7.33 (s, 1H, NH), 7.30 (t, 2H, J_{HH} = 8.2 Hz, Ph-H), 7.12 (t, 1H, J_{HH} = 7.3 Hz, Ph-H), 4.04 (dd, 1H, J_{HH} = 6.1 and 8.2 Hz, CH), 1.96 (s, 3H, CH_3), 1.95 (m, 1H, CH), 1.75 (ddd, 1H, J_{HH} = 2.3, 5.8 and 8.2 Hz, CH_aH_b), 0.99 (d,3H, J_{HH} = 6.6 Hz, CH_3), 0.99 (br. s, 2H, CH_2), 0.95 (d,3H, J_{HH} = 6.6 Hz, CH_3).

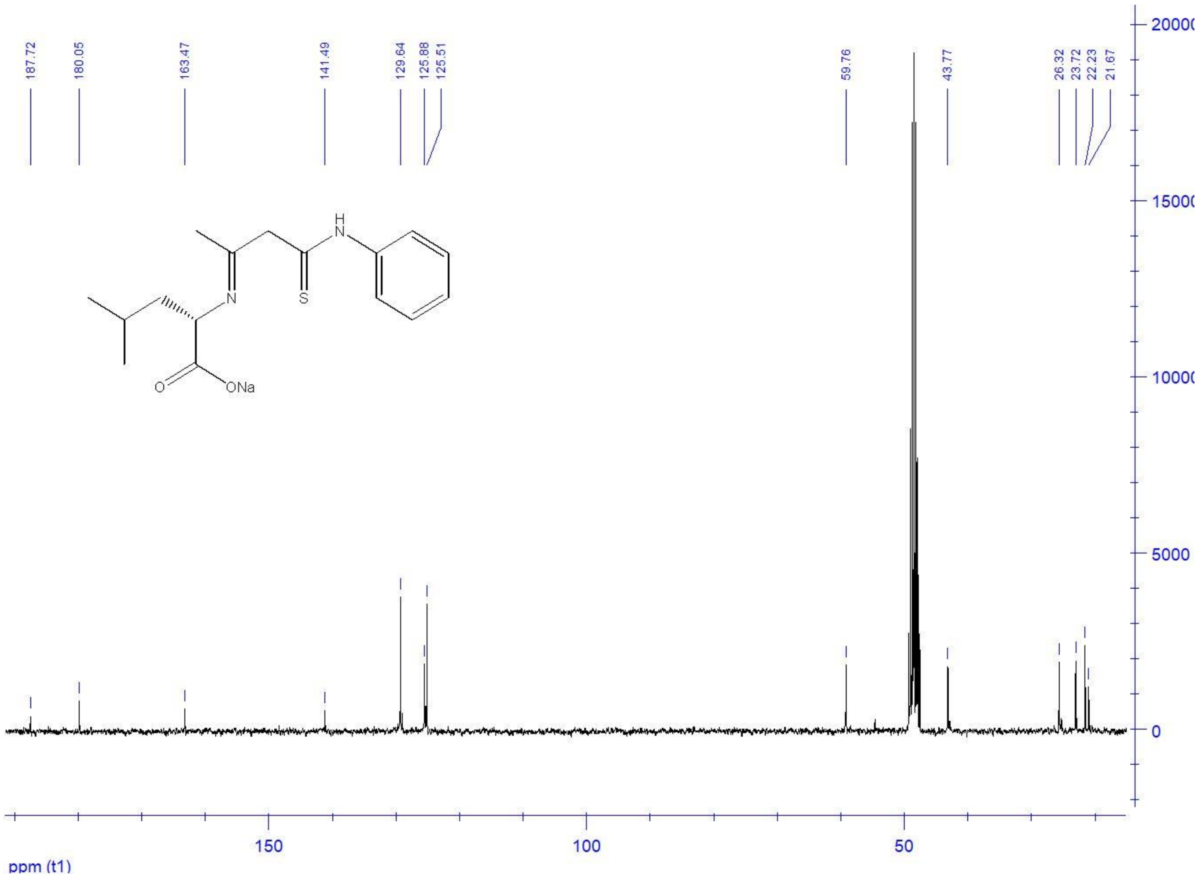

Figure S6. ^{13}C-NMR spectrum of L_3. (CD_3OD). σ [ppm] = 187.7 (C=S), 180.1 (COONa), 163.5 (C=N), 141.5 (Ph), 129.6 (Ph), 125.9 (Ph), 125.5 (Ph), 59.8 (CH), 43.8 (CH_2), 26.3 (CH_2), 23.7 (CH), 22.2 (CH_3), 21.7 (CH_3).

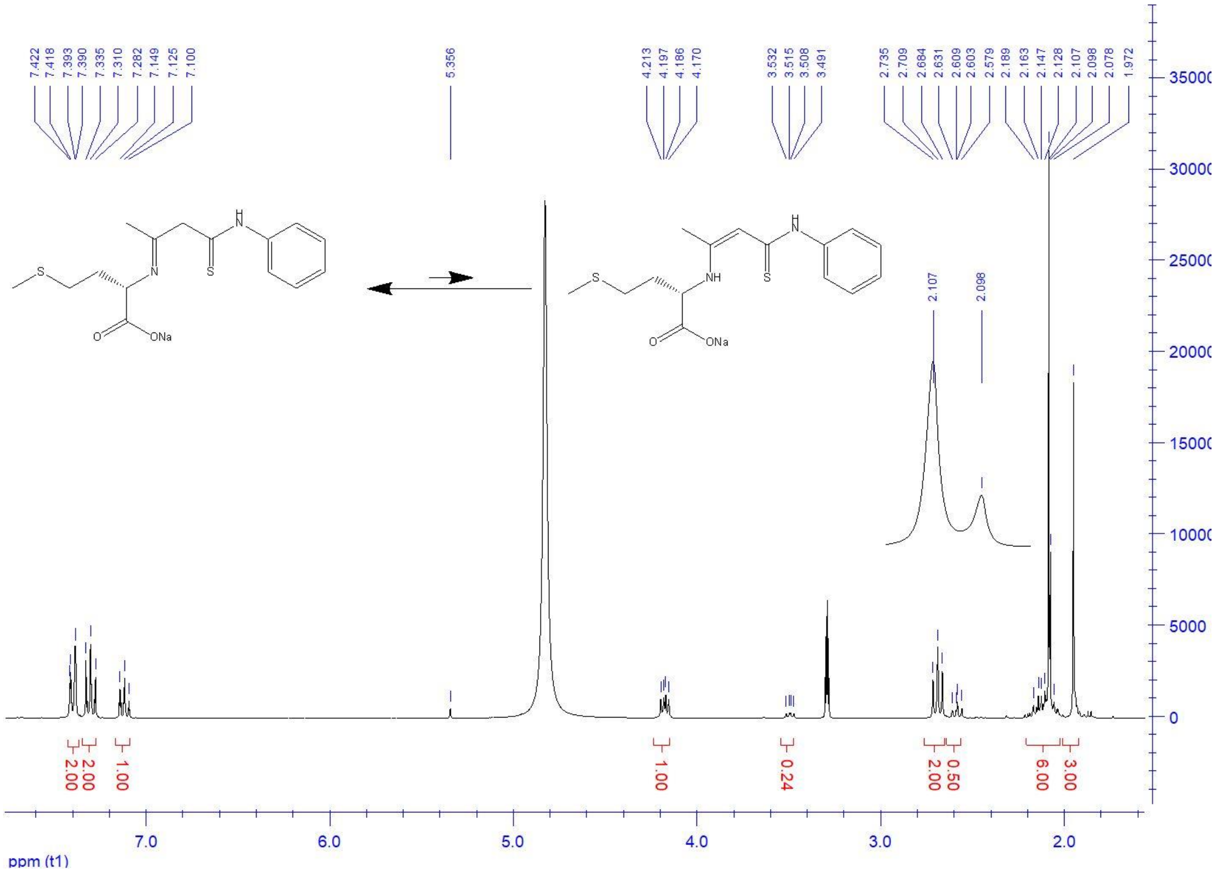

Figure S7. ^{1}H-NMR spectrum of L_4. (CD_3OD). σ [ppm] = 7.41 (d, 2H, J_{HH} = 8.3 Hz, Ph-H), 7.31 (t, 2H, J_{HH} = 8.3 Hz, Ph-H), 7.13 (t, 1H, J_{HH} = 7.3 Hz, Ph-H), 5.36 (s, =CH enamine), 4.19 (dd, 1H, J_{HH} = 4.8 and 8.1 Hz, CH imine), 3.51 (dd, J_{HH} = 5.2 and 7.3 Hz CH enamine), 2.71 (t, 2H, J_{HH} = 7.4 Hz, SCH_2 imine), 2.60 (dd, J_{HH} = 6.8 and 8.8 Hz, SCH_2 enamine), 2.15 (m, 3H, CH and CH_2), 2.11 (s, 3H, SCH_3 imine), 2.10 (s, SCH_3 enamine), 1.97 (s, 3H, CH_3).

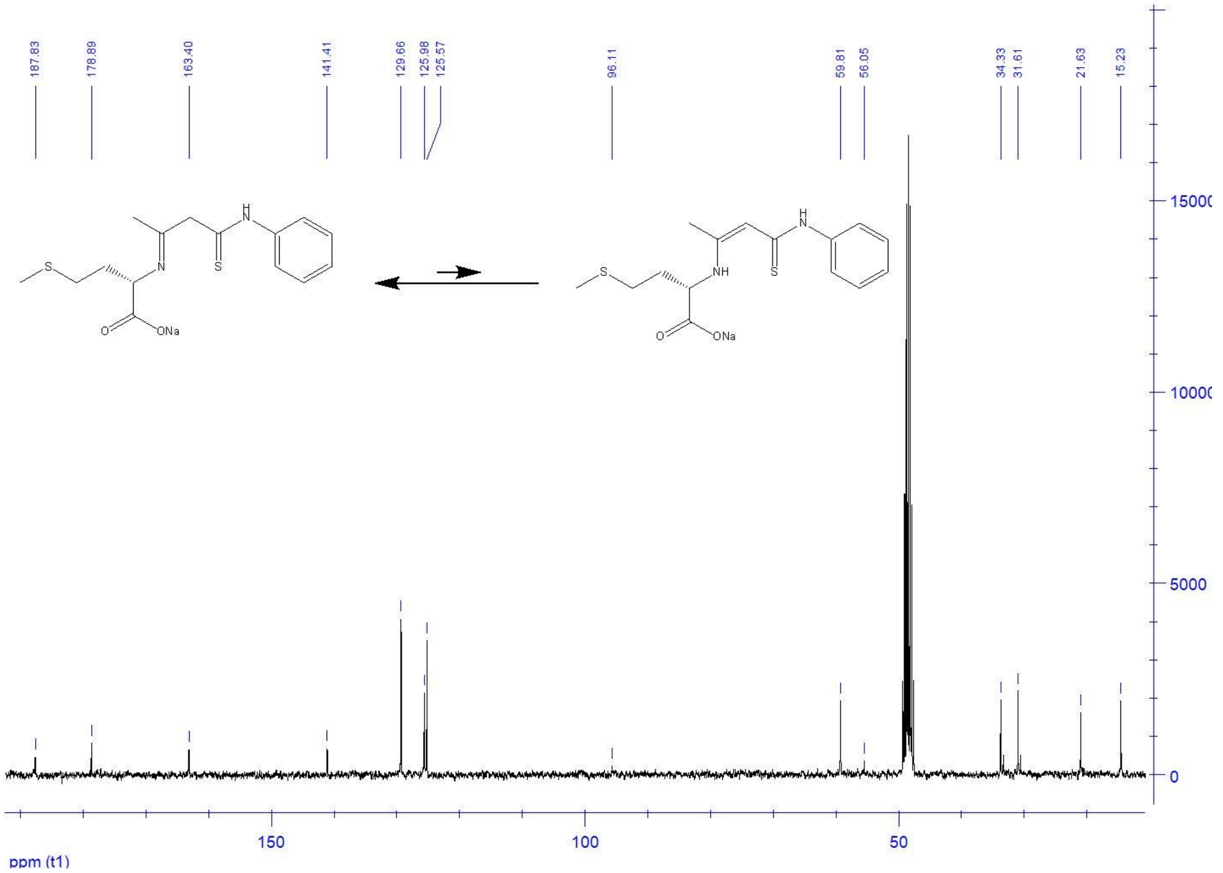

Figure S8. ^{13}C-NMR spectrum of L_4. (CD_3OD). σ [ppm] = 187.8 (C=S), 178.9 (COONa), 163.4 C=N), 141.4 (Ph), 129.7 (Ph), 126.0 (Ph), 125.6 (Ph), 96.1 (=CH enamine), 59.8 (CH_2), 56.1 (CH), 34.3 (SCH_2), 31.6 (CH_2), 21.6 (CH_3), 15.2 (SCH_3).

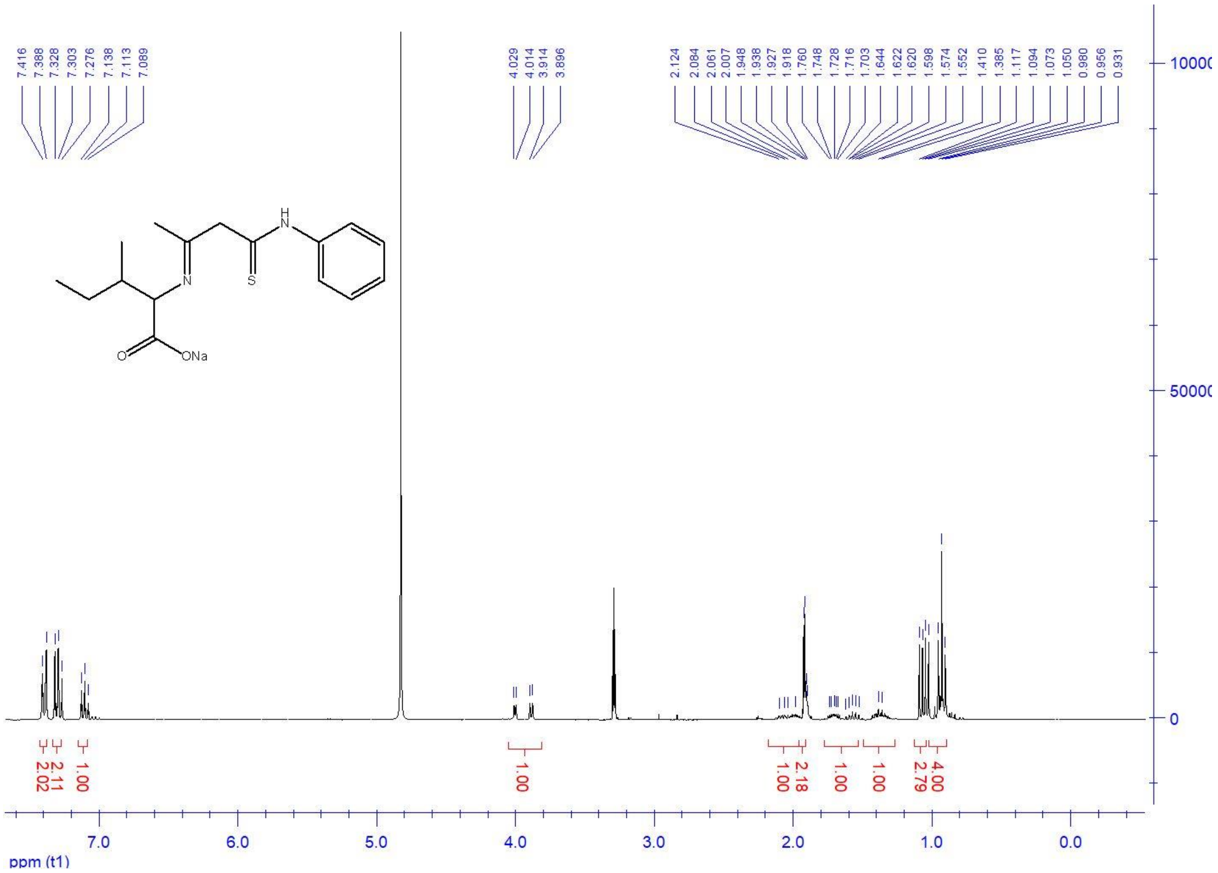

Figure S9. ^{1}H-NMR spectrum of L_5. (CD_3OD). σ [ppm] = 7.40 (d, 2H, J_{HH} = 7.3 Hz, Ph-H), 7.30 (t, 2H, J_{HH} = 8.2 Hz, Ph-H), 7.11 (t, 1H, J_{HH} = 7.3 Hz, Ph-H), 4.02 and 3.91 (d, 1H, J_{HH} = 4.3 and 5.6 Hz, CH), 2.07 (m, 1H, CH), 1.95 and 1.94 (s, 3H, CH_3), 1.64 (m, 1H, CH_aH_b), 1.39 (m, 1H, CH), 1.10 and 1.06 (d, 3H, J_{HH} = 6.9 and 6.8 Hz, CH_3), 0.96 (t, 3H, J_{HH} = 7.4 Hz, CH_3).

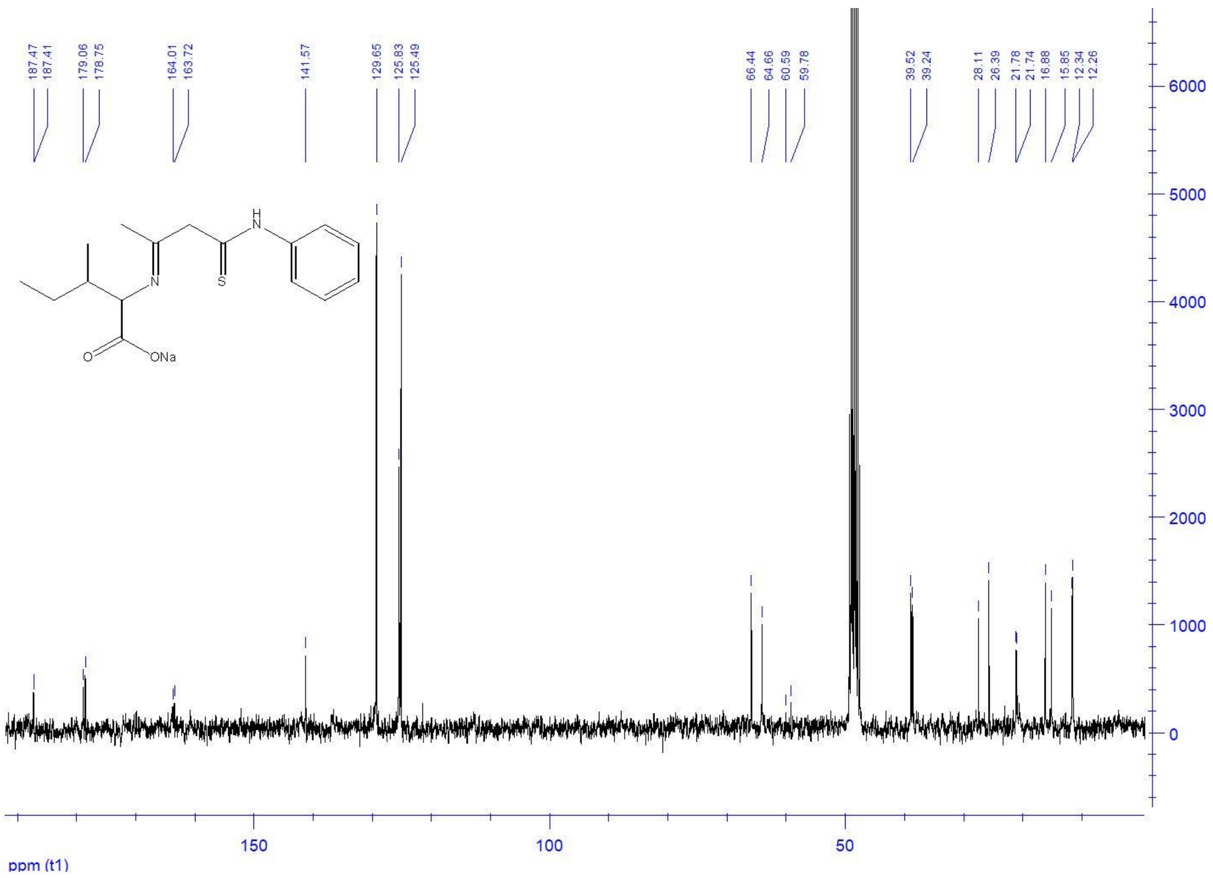

Figure S10. ^{13}C-NMR spectrum of L_5. (CD_3OD). σ [ppm] = 187.5 and 187.4 (C=S), 179.1 and 178.8 (COONa), 164.0 and 163.7 (=CN), 141.6 (Ph), 129.6 (Ph), 125.8 (Ph), 125.5 (Ph), 66.4 and 64.7 (CH_2), 60.6 and 59.8 (CH), 39.5 and 39.2 (CH), 28.1 and 26.4 (CH_2), 21.7 and 21.7 (CH_3), 16.9 and 15.8 (CH_3), 12.3 and 12.2 (CH_3).

IR spectra of ligands L_1-L_5

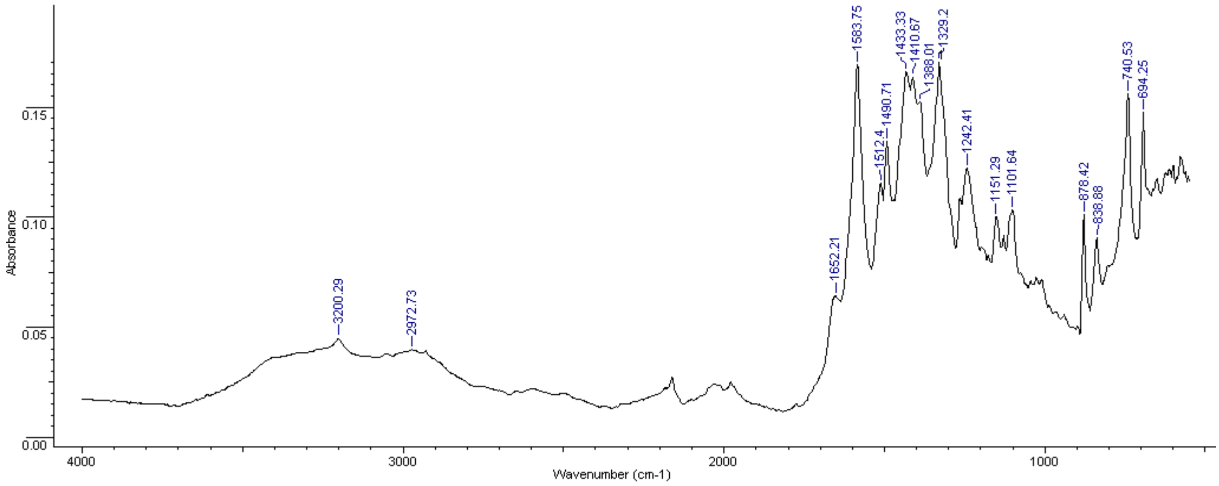

Figure S11. IR-ATR spectrum of L_1

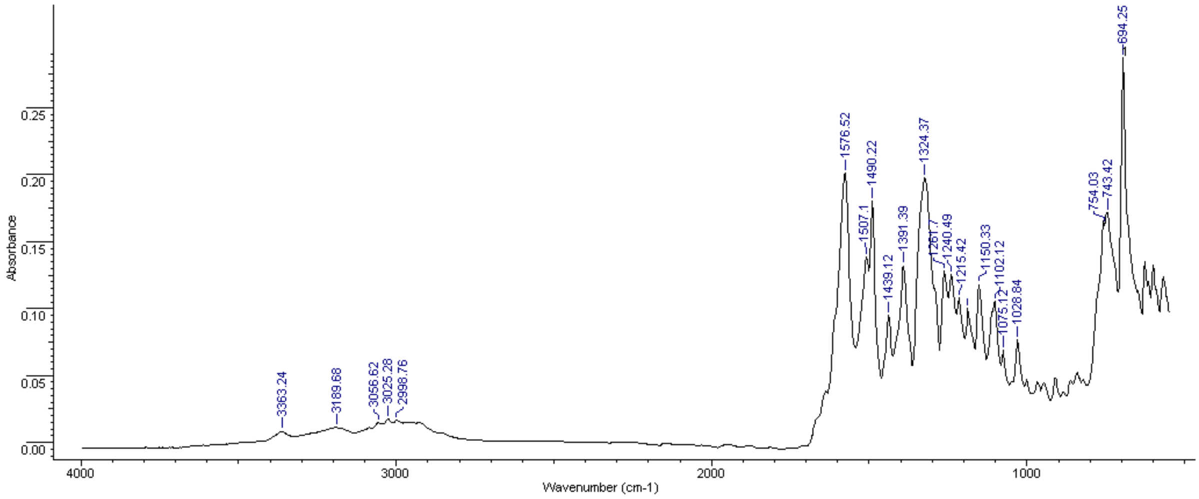

Figure S12. IR-ATR spectrum of L_2

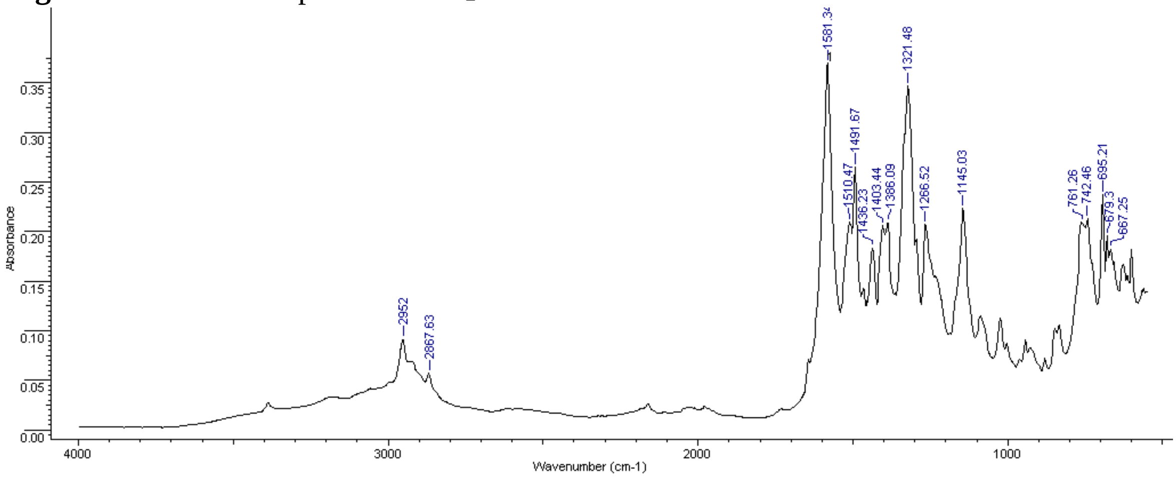

Figure S13. IR-ATR spectrum of L_3

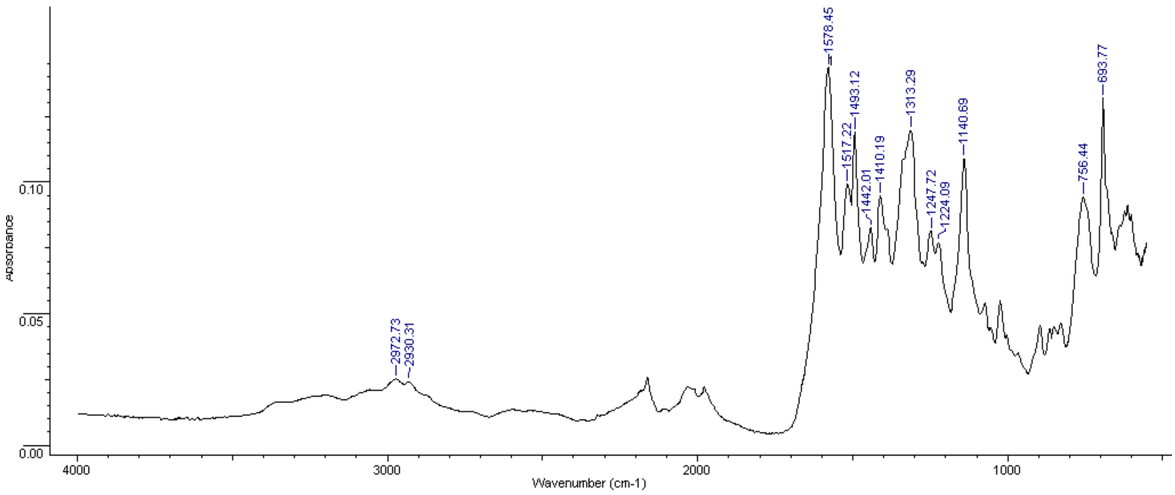

Figure S14. IR-ATR spectrum of L_4

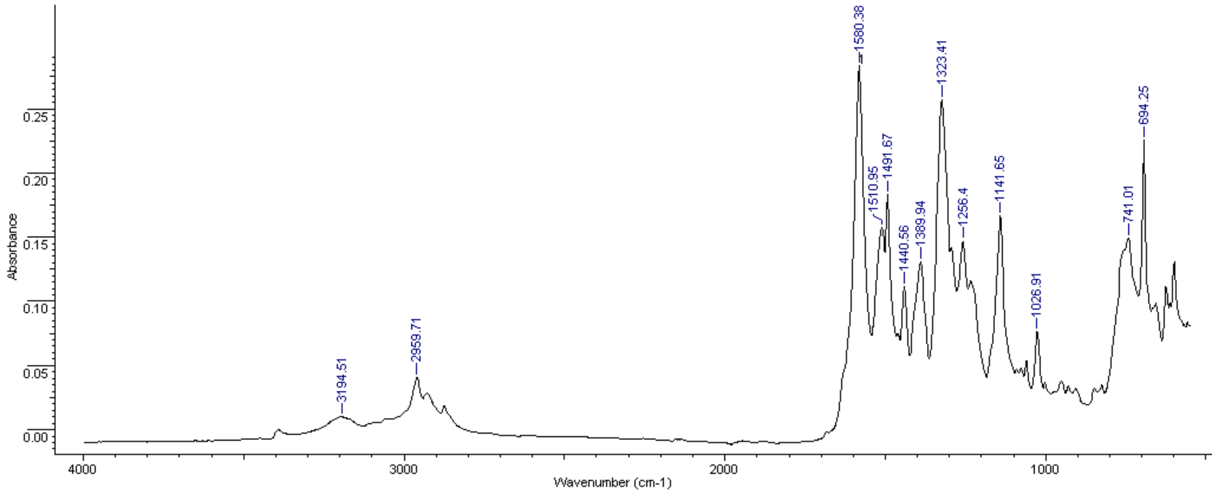

Figure S15. IR-ATR spectrum of L_5

IR spectra of complexes.

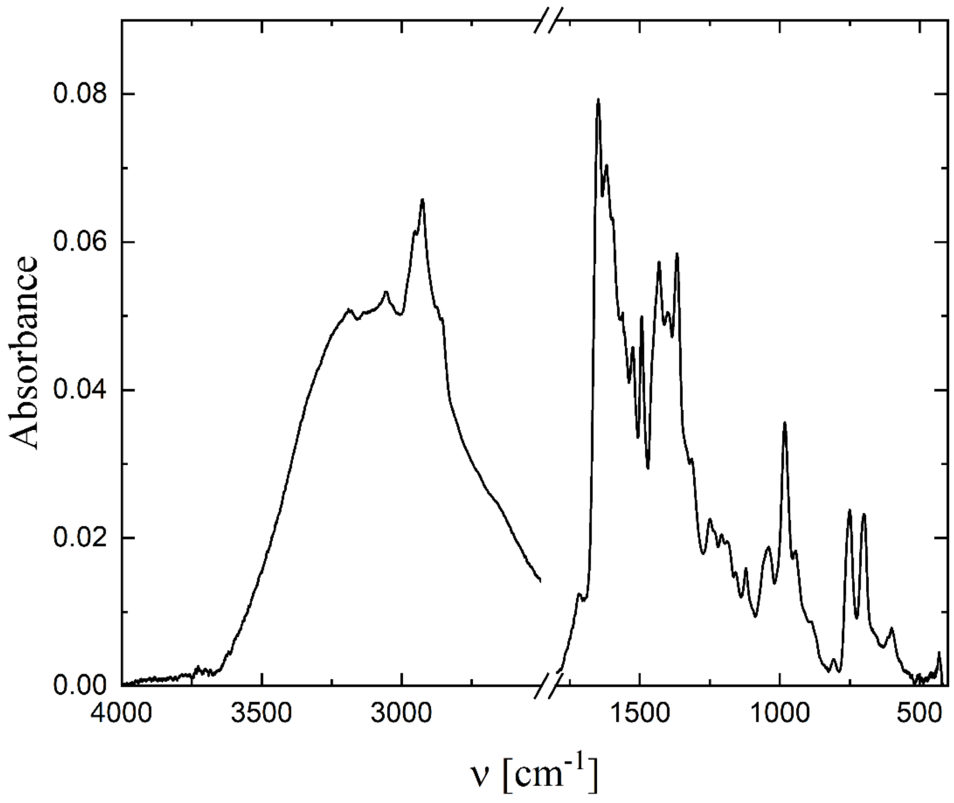

Figure S16. IR-ATR spectrum of **VC054**.

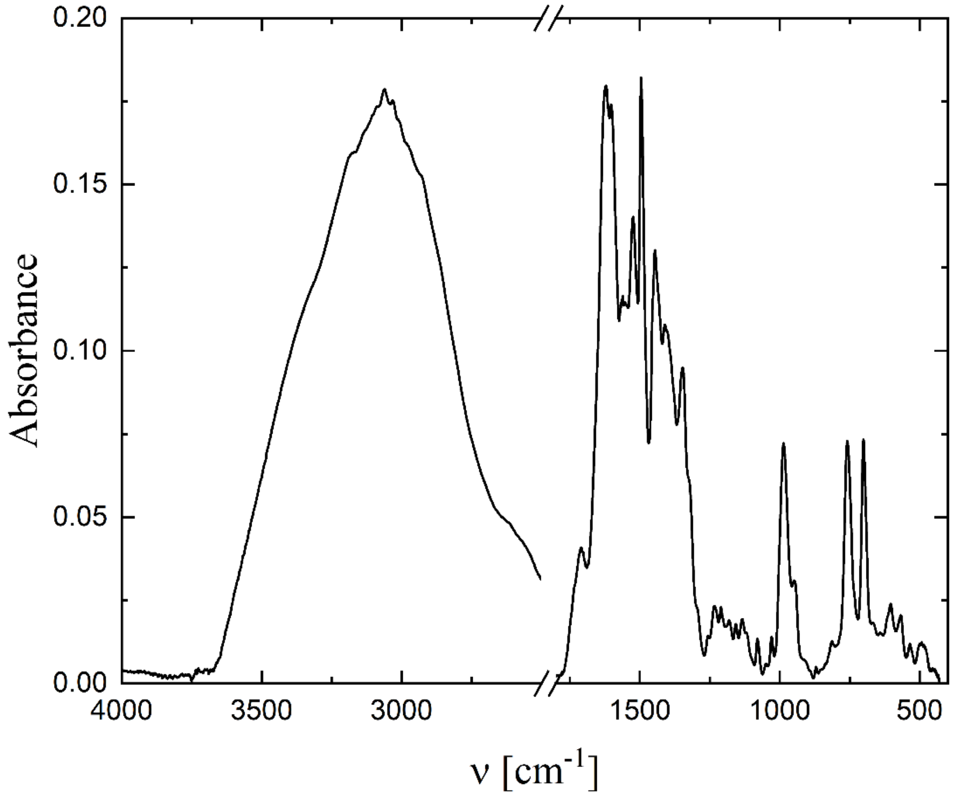

Figure S17. IR-ATR spectrum of **VC059**.

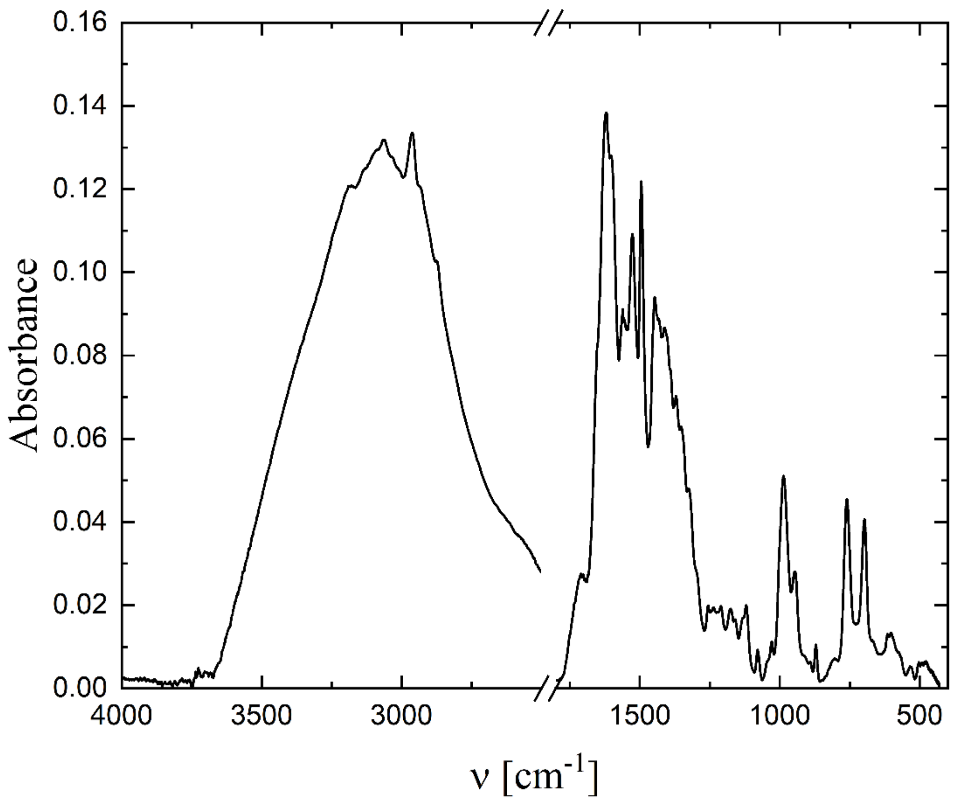

Figure S18. IR-ATR spectrum of **VC070**.

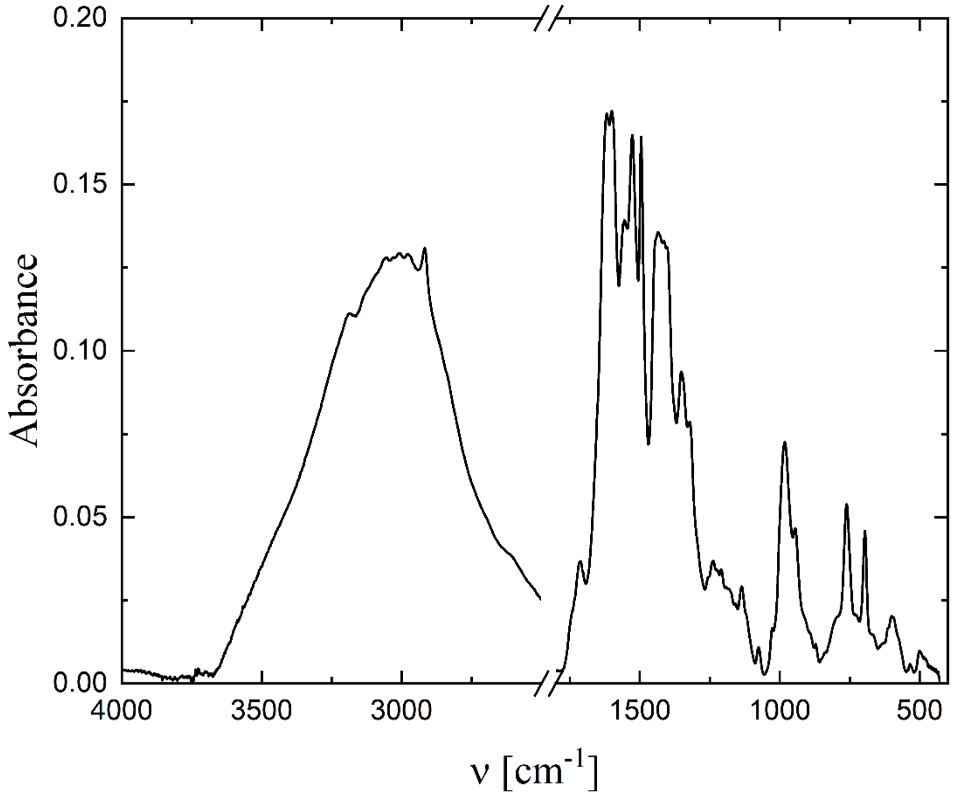

Figure S19. IR-ATR spectrum of **VC073**.

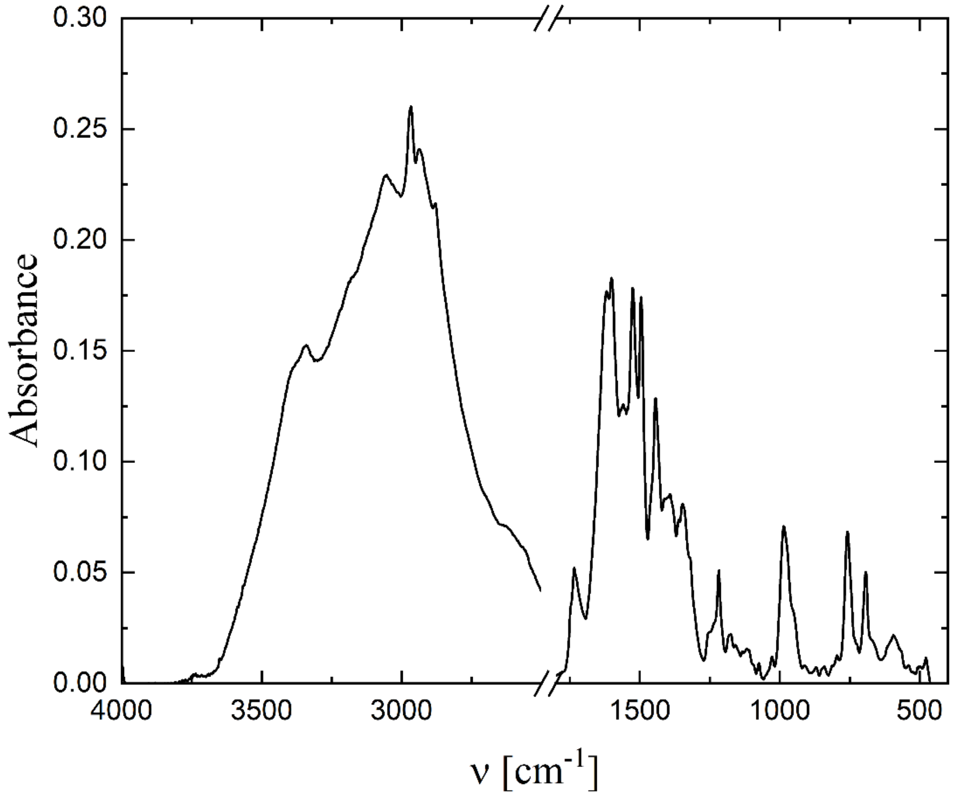

Figure S20. IR-ATR spectrum of **VC109**.

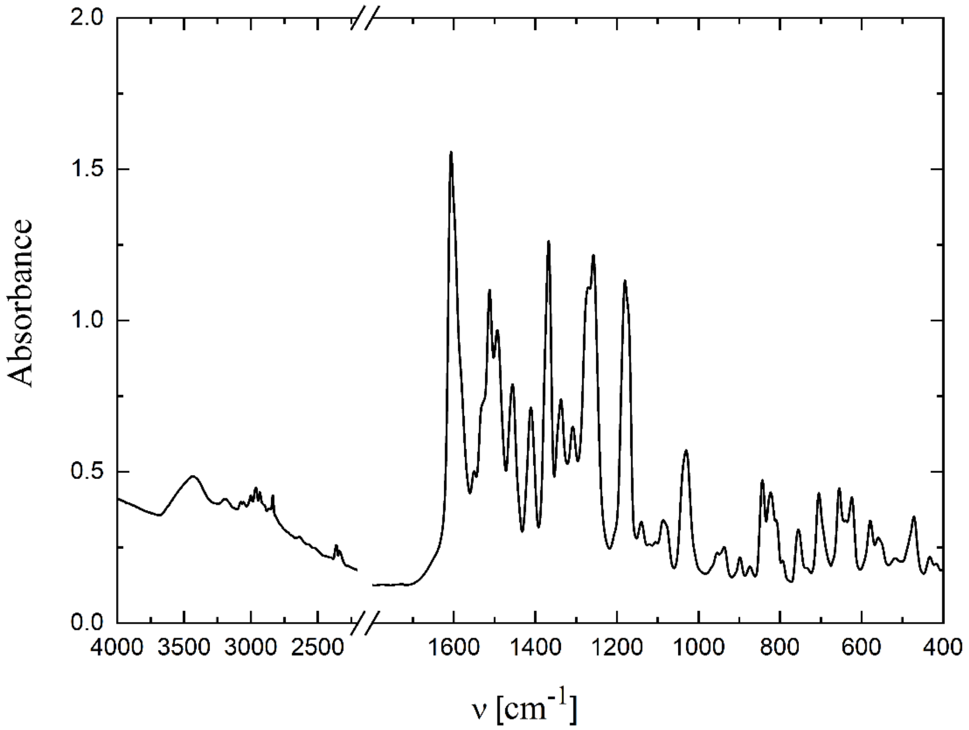

Figure S21. IR-ATR spectrum of **VC055**.

UV-Vis spectra of complexes

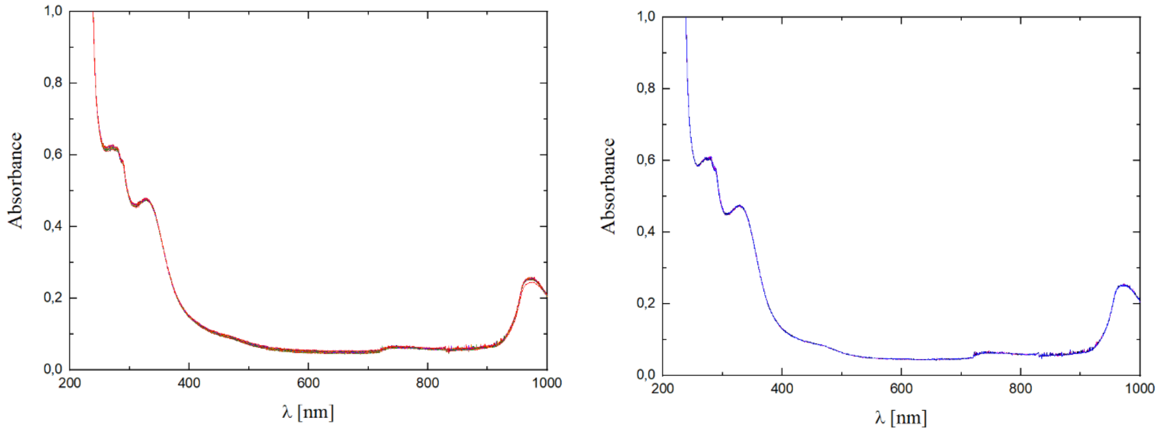

Figure S22. UV–Vis spectra of complex **VC054** in DMSO-H_2O mixture (20 µl + 3 ml respectively) at pH = 7 (left side) and pH = 2 (right side). T = 37 °C, d = 1 cm, 15 spectra measured in 340 s intervals.

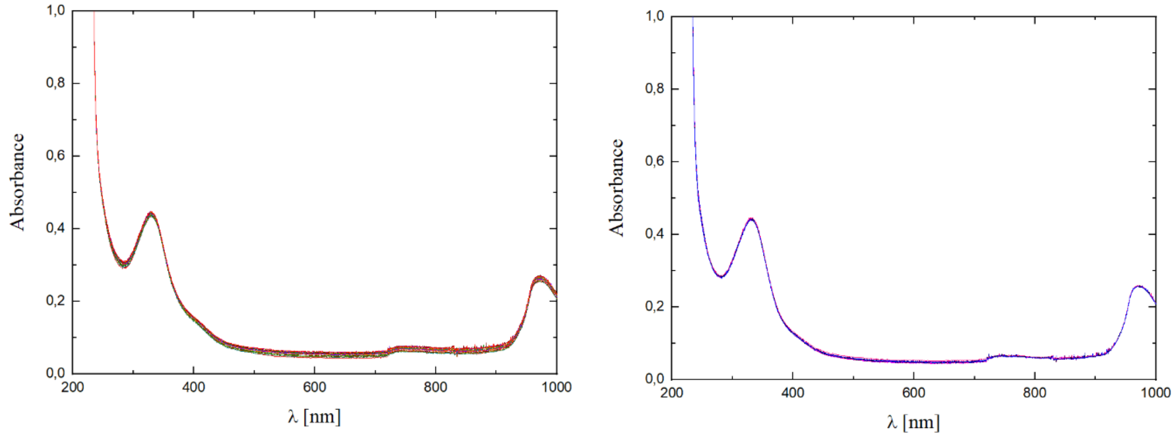

Figure S23. UV–Vis spectra of complex **VC059** in DMSO-H_2O mixture (20 µl + 3 ml respectively) at pH = 7 (left side) and pH = 2 (right side). T = 37 °C, d = 1 cm, 15 spectra measured in 340 s intervals.

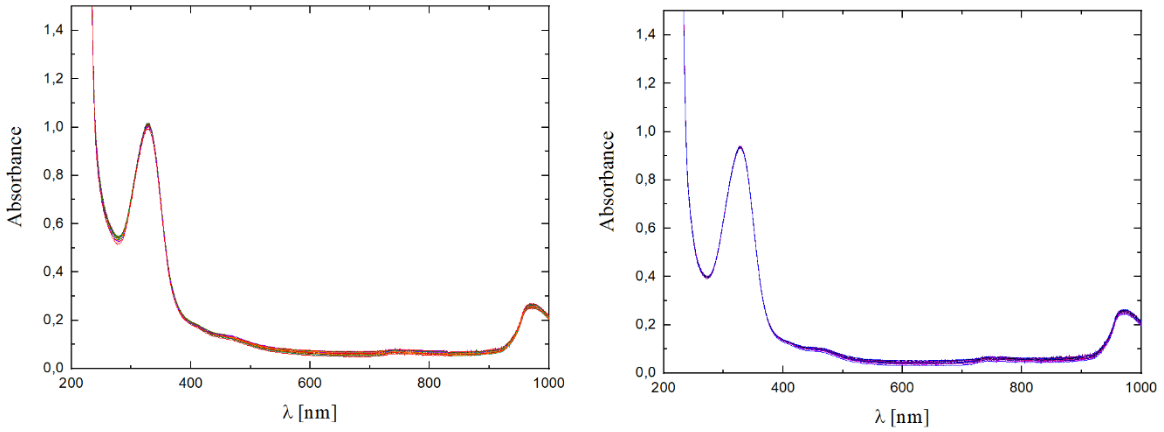

Figure S24. UV–Vis spectra of complex **VC070** in DMSO-H_2O mixture (20 µl + 3 ml respectively) at pH = 7 (left side) and pH = 2 (right side). T = 37 °C, d = 1 cm, 15 spectra measured in 340 s intervals.

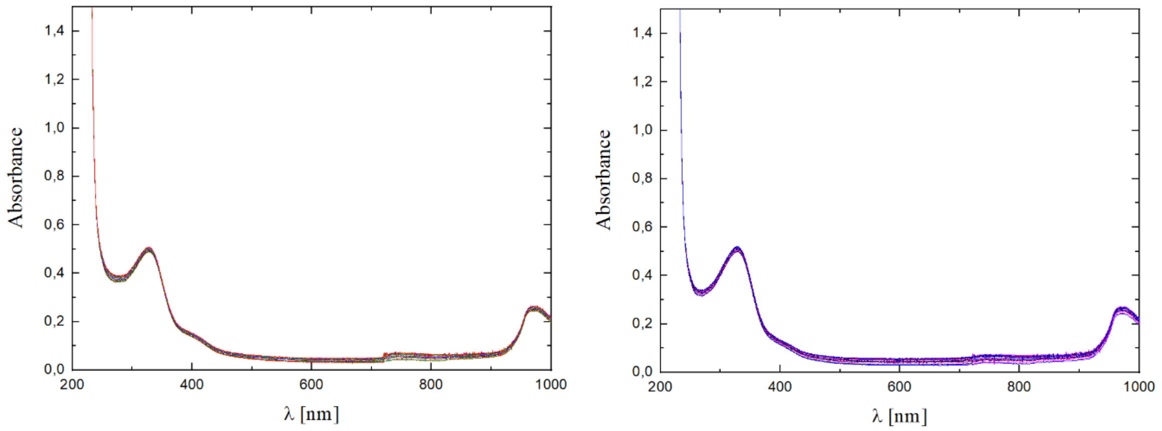

Figure S25. UV–Vis spectra of complex **VC073** in DMSO-H_2O mixture (20 µl + 3 ml respectively) at pH = 7 (left side) and pH = 2 (right side). T = 37 °C, d = 1 cm, 15 spectra measured in 340 s intervals.

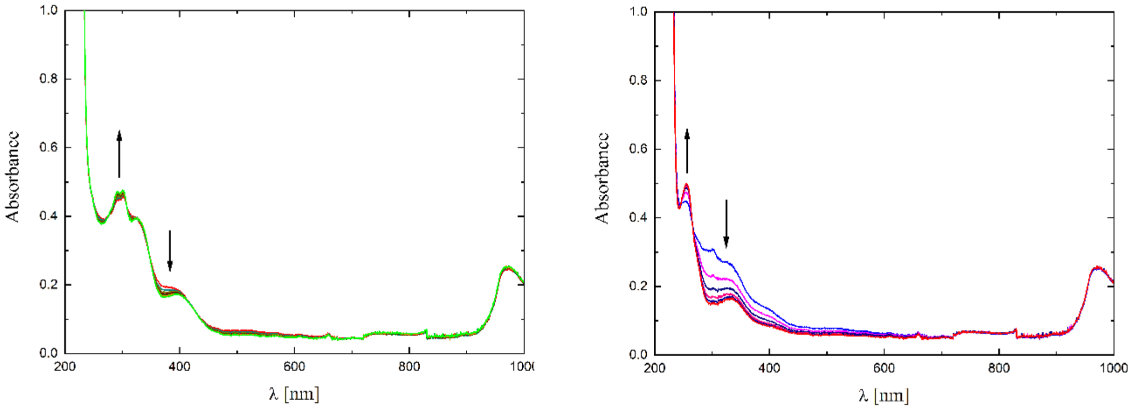

Figure S26. UV–Vis spectra of complex **VC055** in DMSO-H_2O mixture (20 µl + 3 ml respectively) at pH = 7 (left side) and pH = 2 (right side). T = 37 °C, d = 1 cm, 7 spectra measured in 340 s intervals. The arrows show direction of changes in spectra.

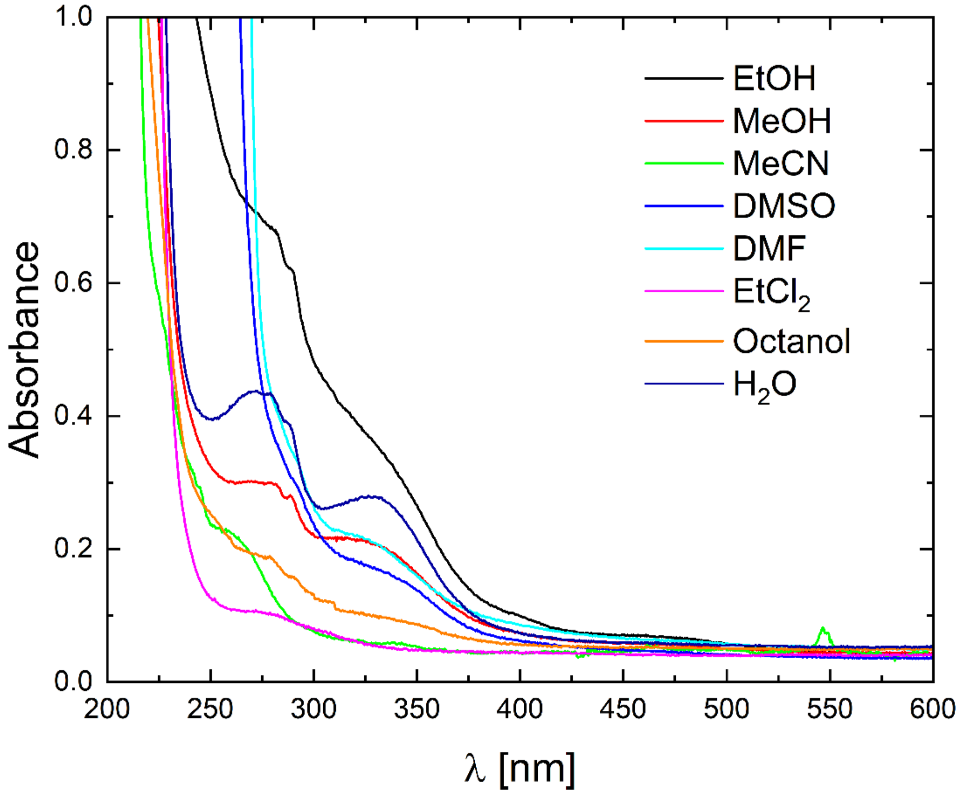

Figure S27. Qualitative UV–Vis spectra of complex **VC054** in different solvents; d = 1 cm.

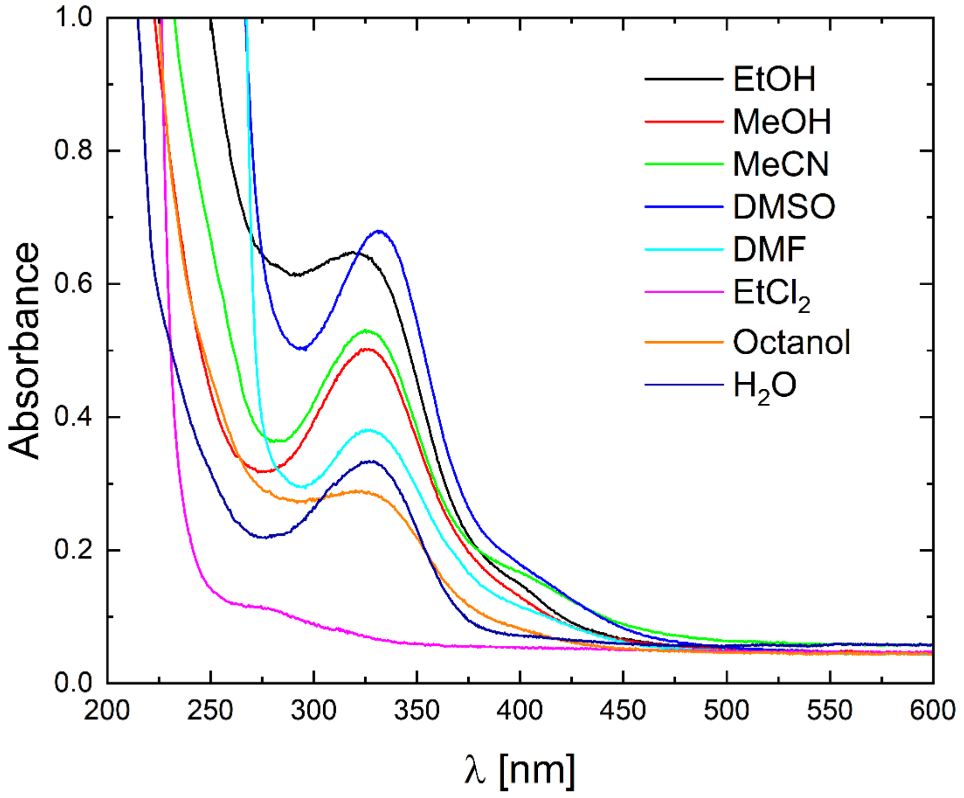

Figure S28. Qualitative UV–Vis spectra of complex **VC059** in different solvents; d = 1 cm.

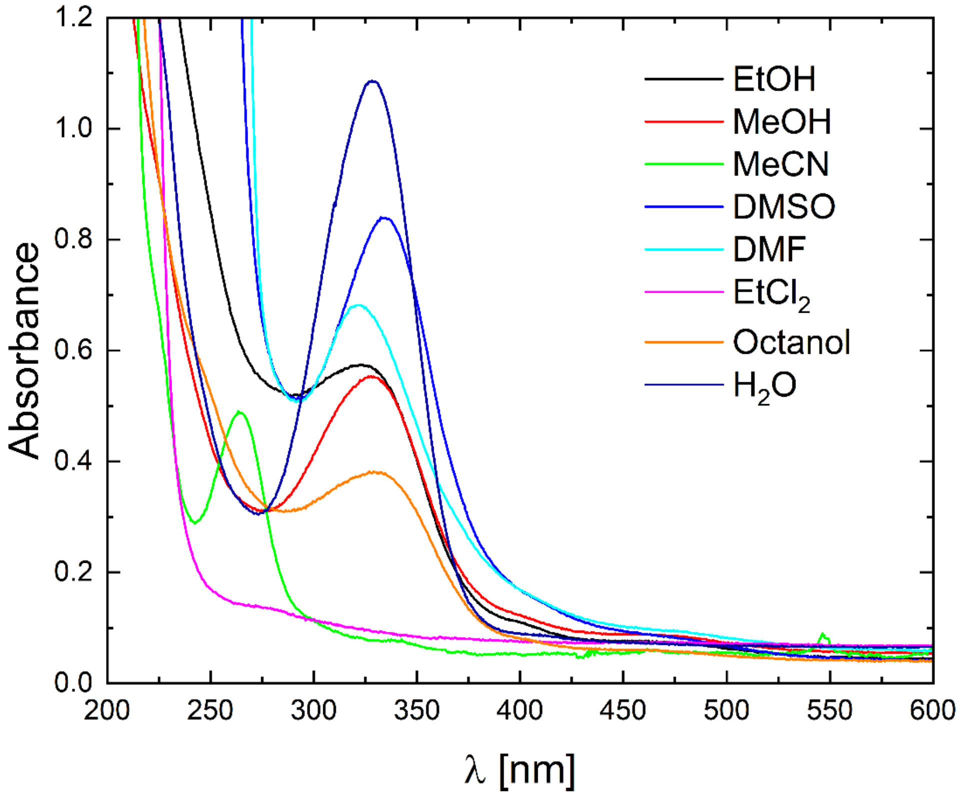

Figure S29. Qualitative UV–Vis spectra of complex **VC070** in different solvents; d = 1 cm.

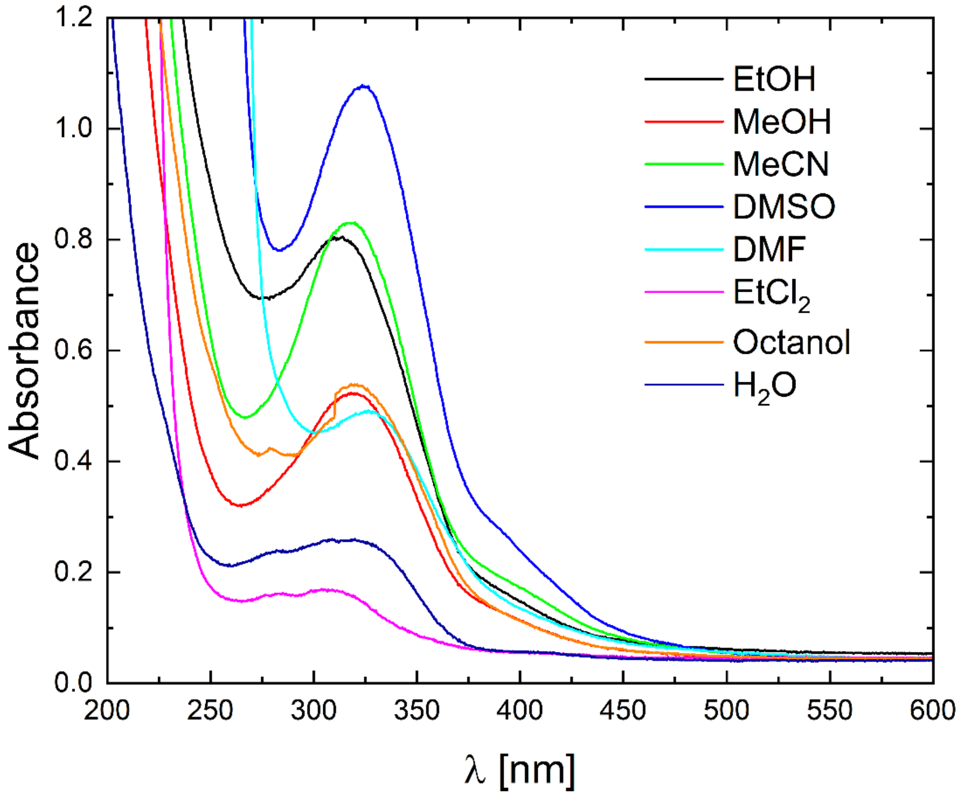

Figure S30. Qualitative UV–Vis spectra of complex **VC073** in different solvents; d = 1 cm.

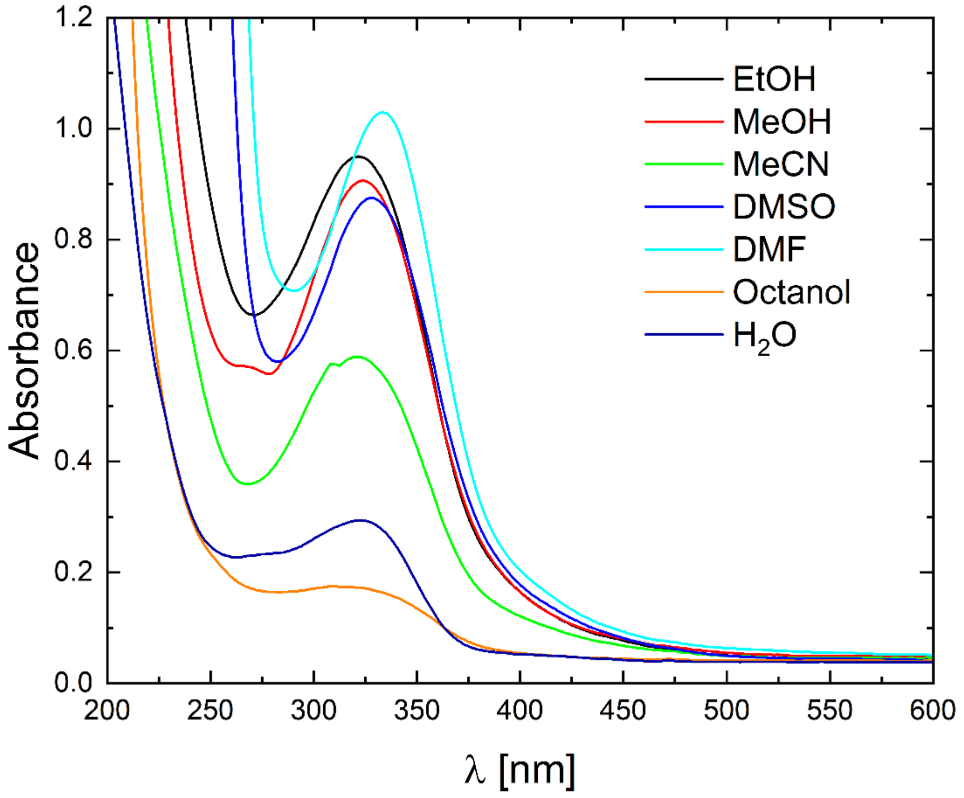

Figure S31. Qualitative UV–Vis spectra of complex **VC109** in different solvents; d = 1 cm.

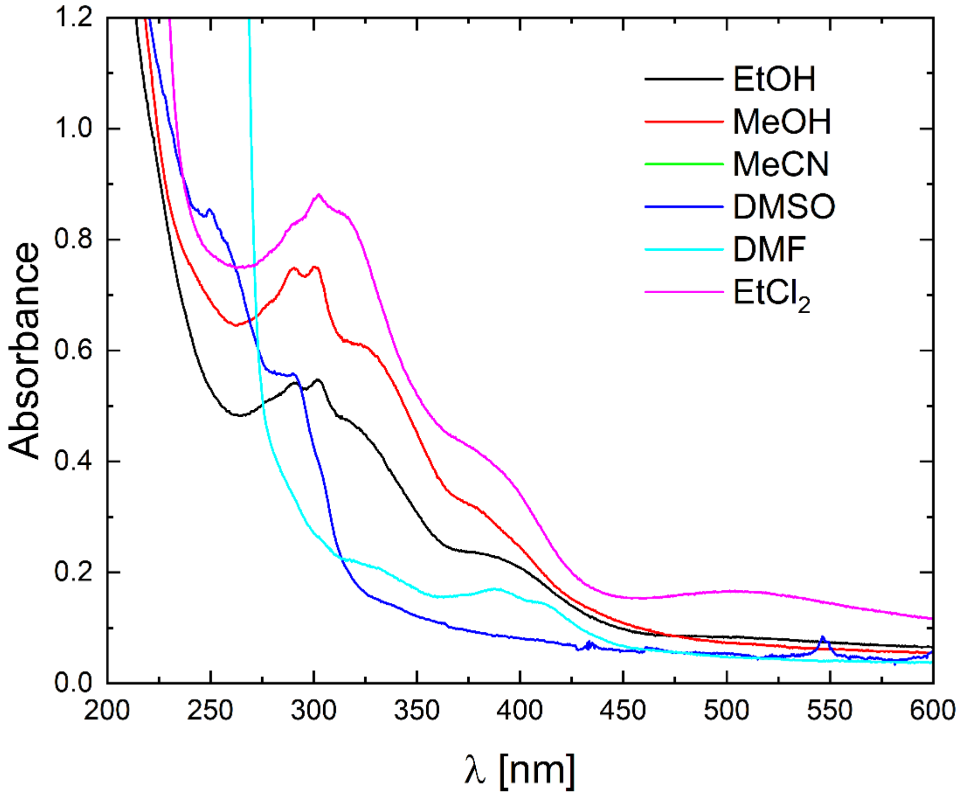

Figure S32. Qualitative UV–Vis spectra of complex **VC055** in different solvents; d = 1 cm.
